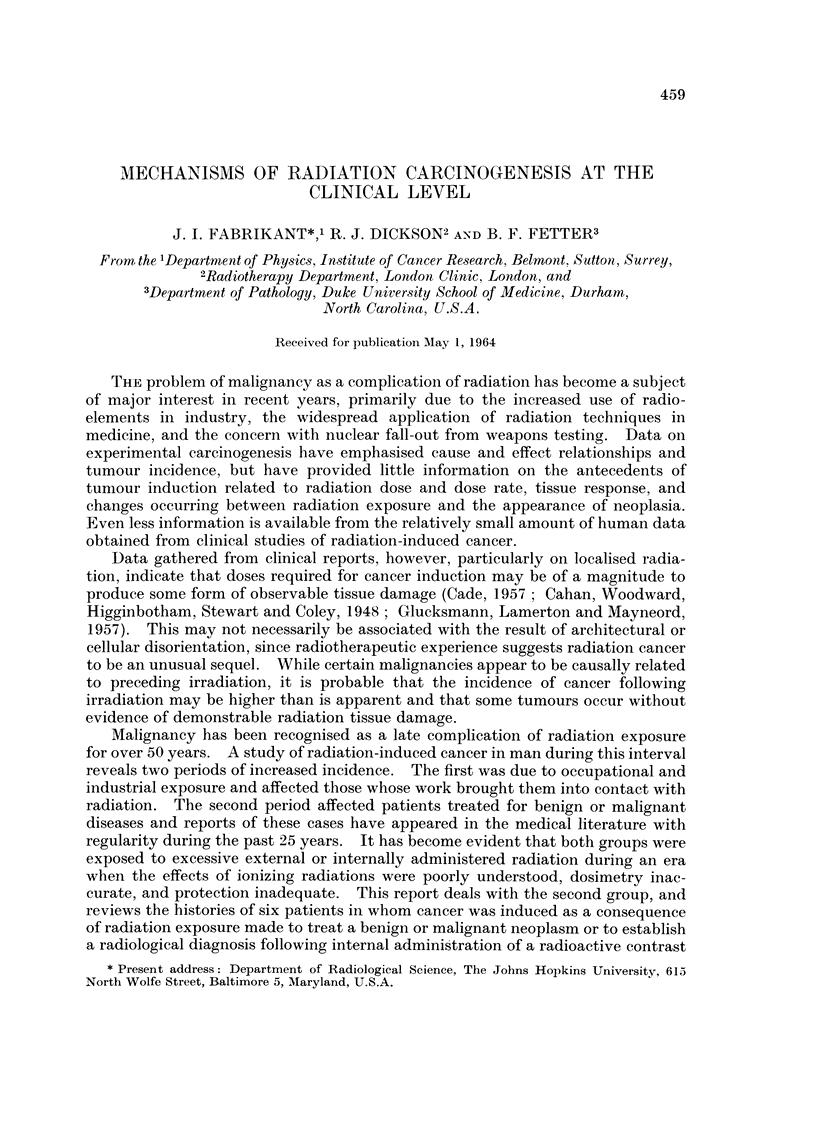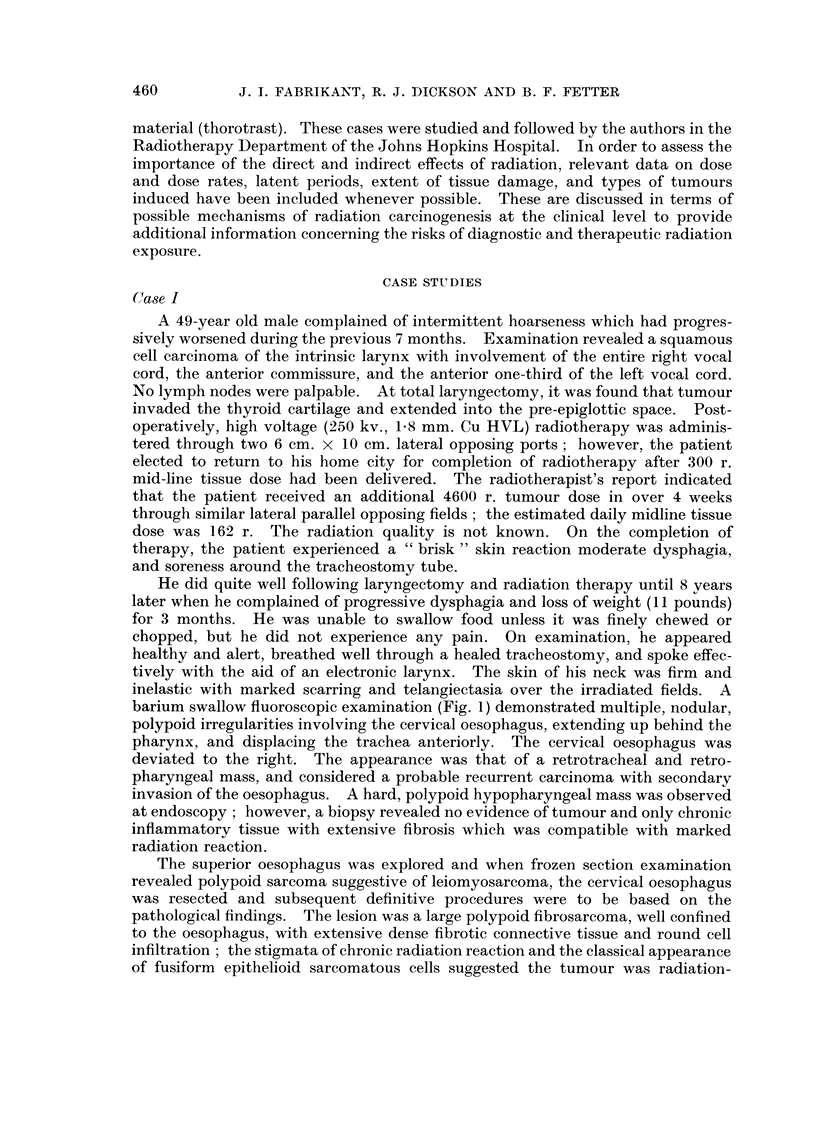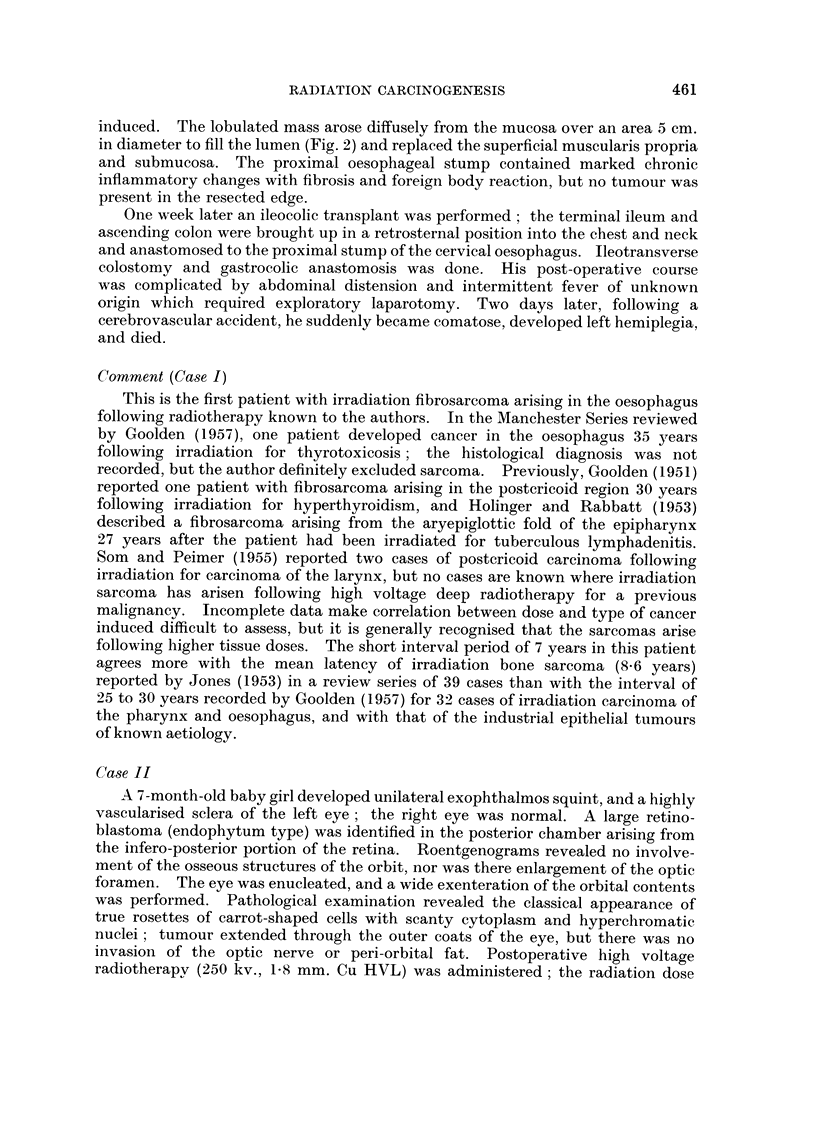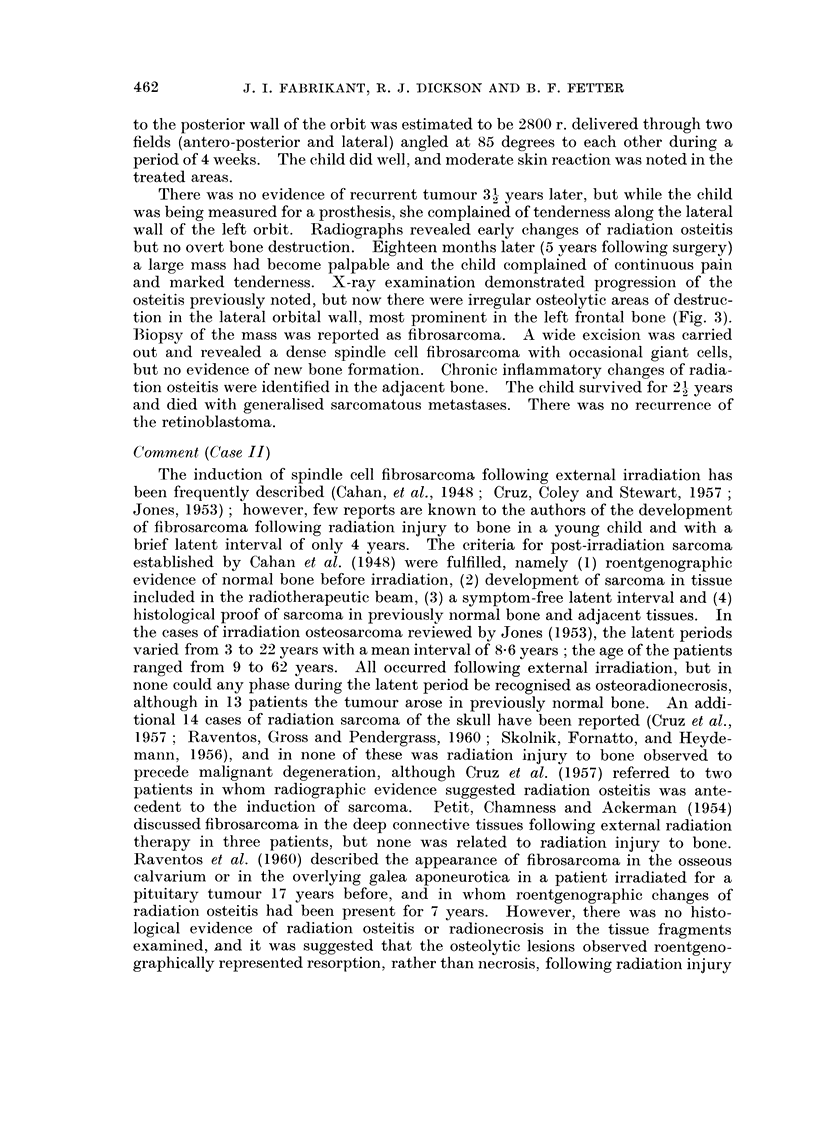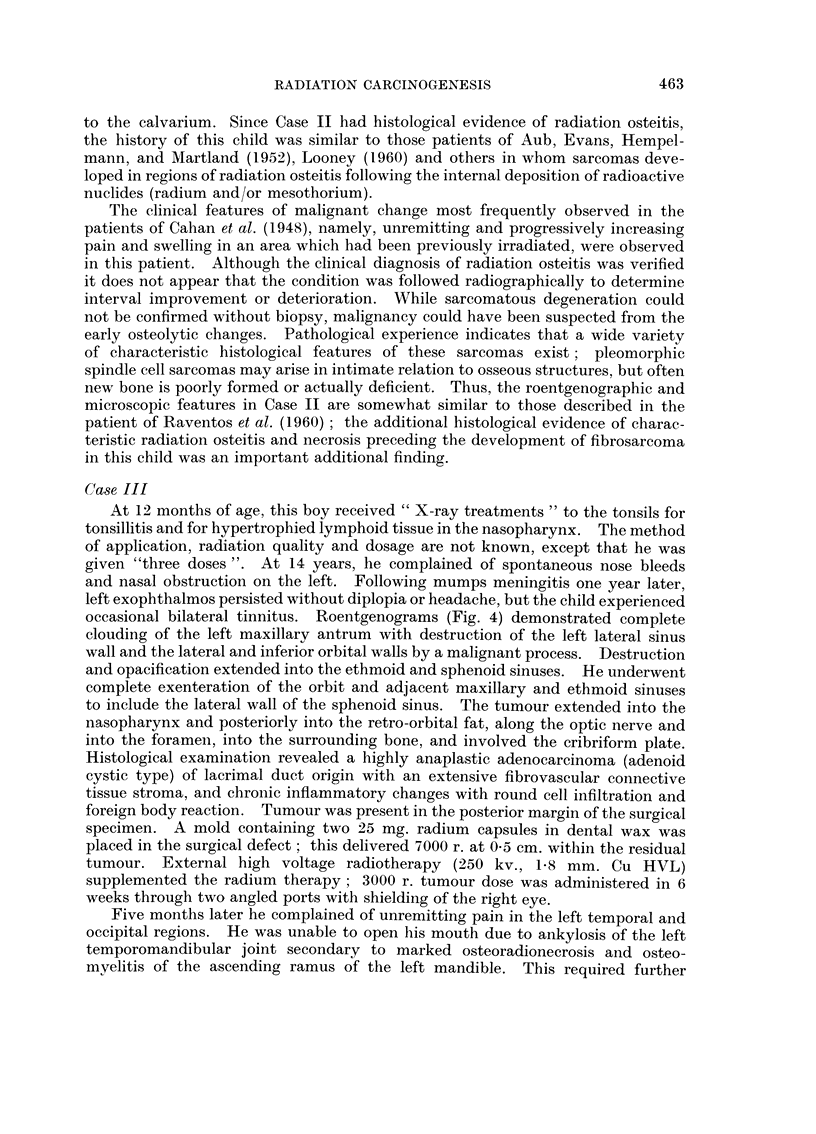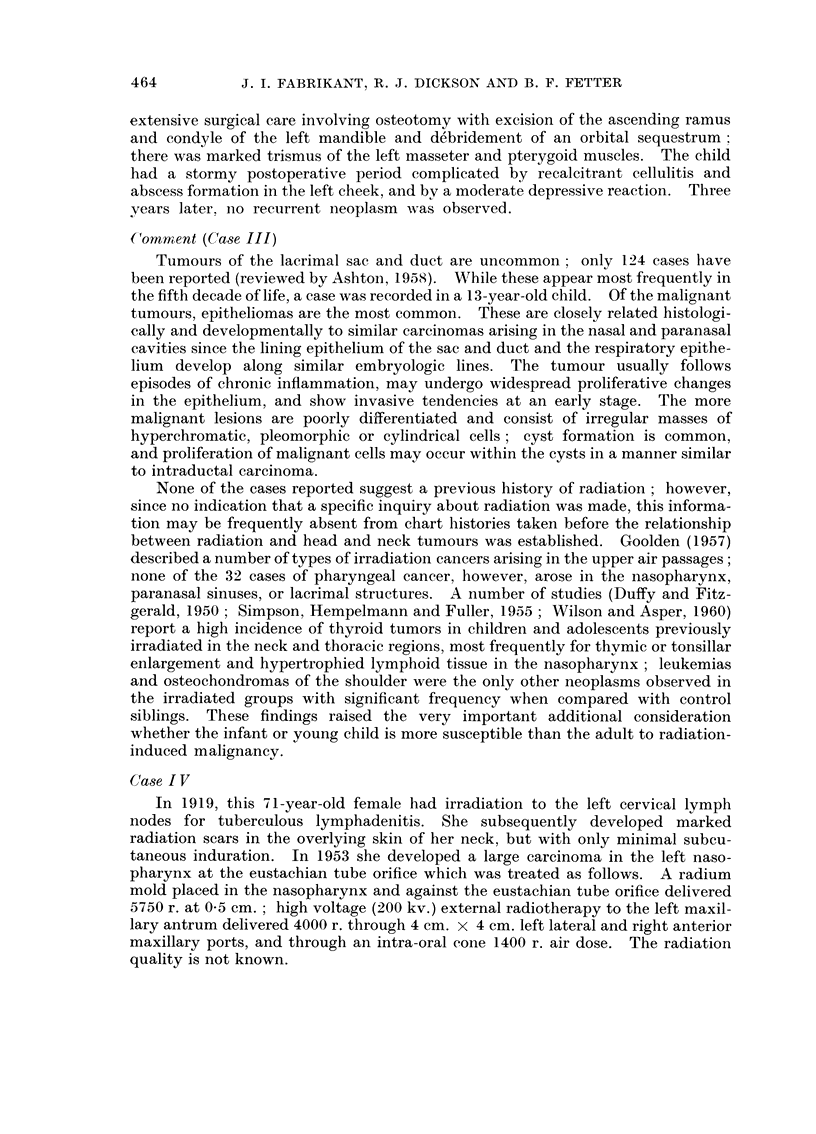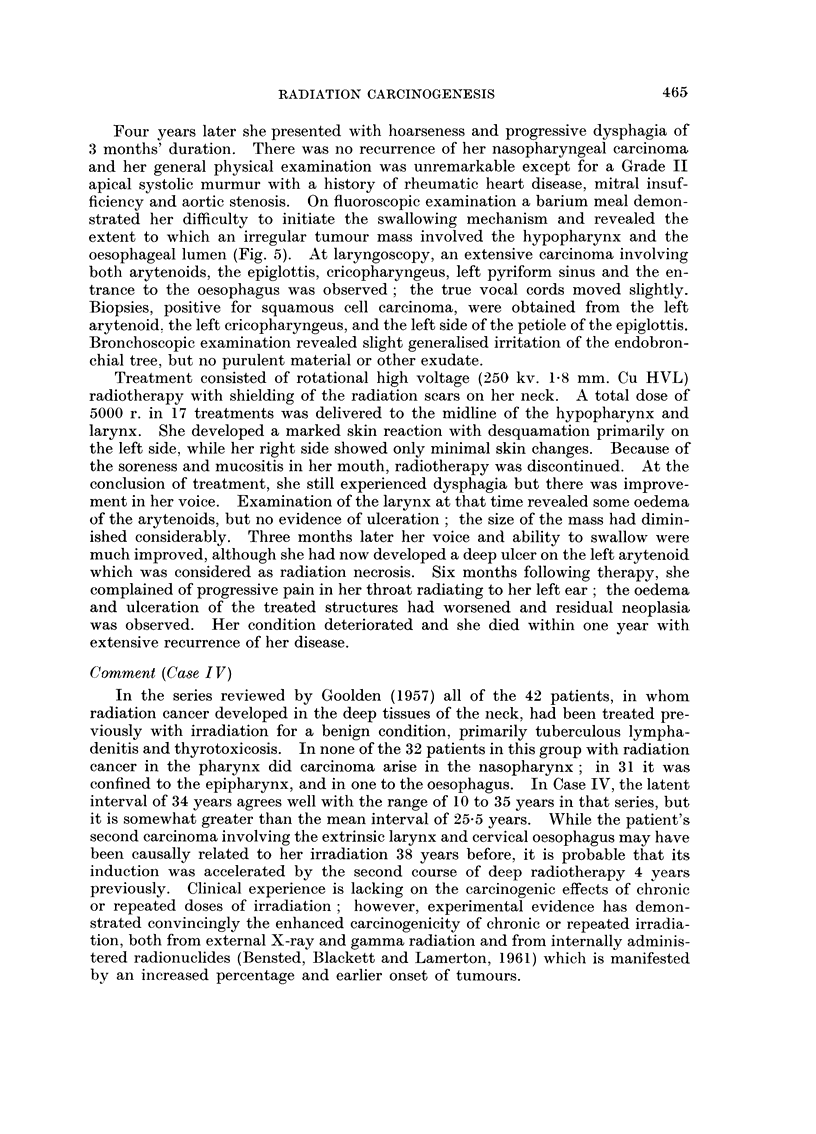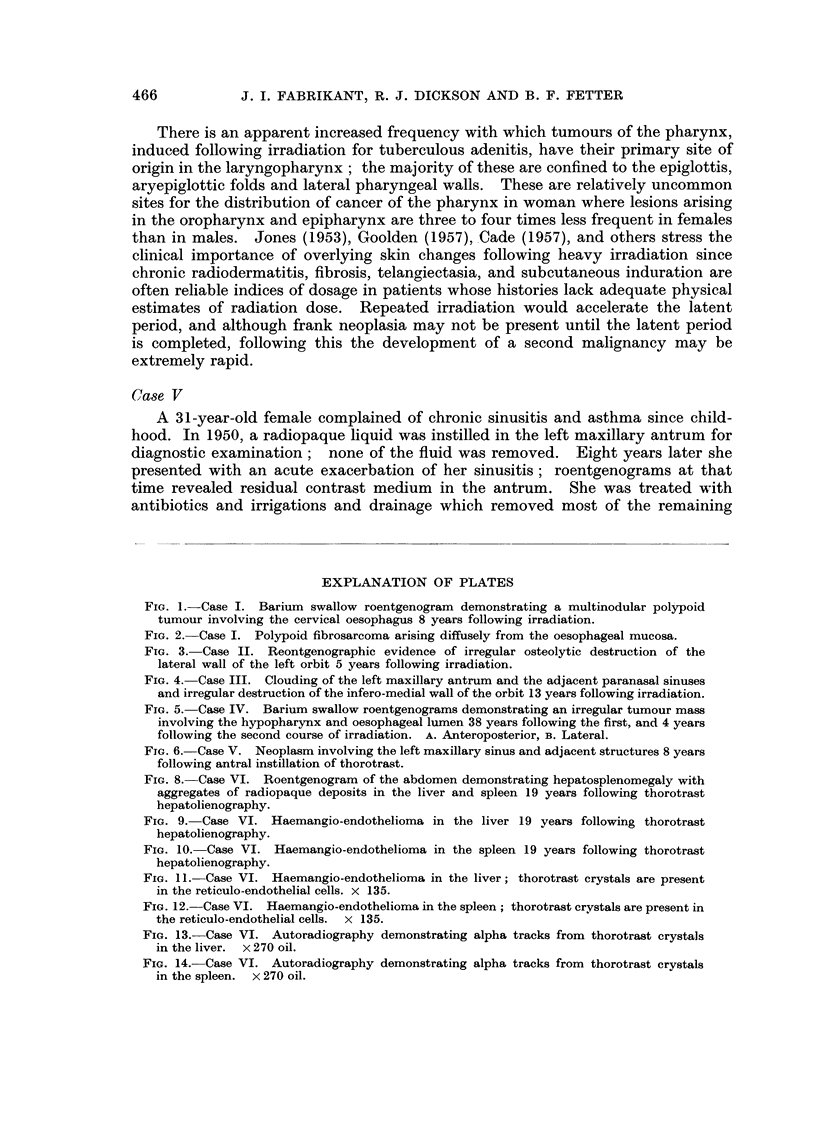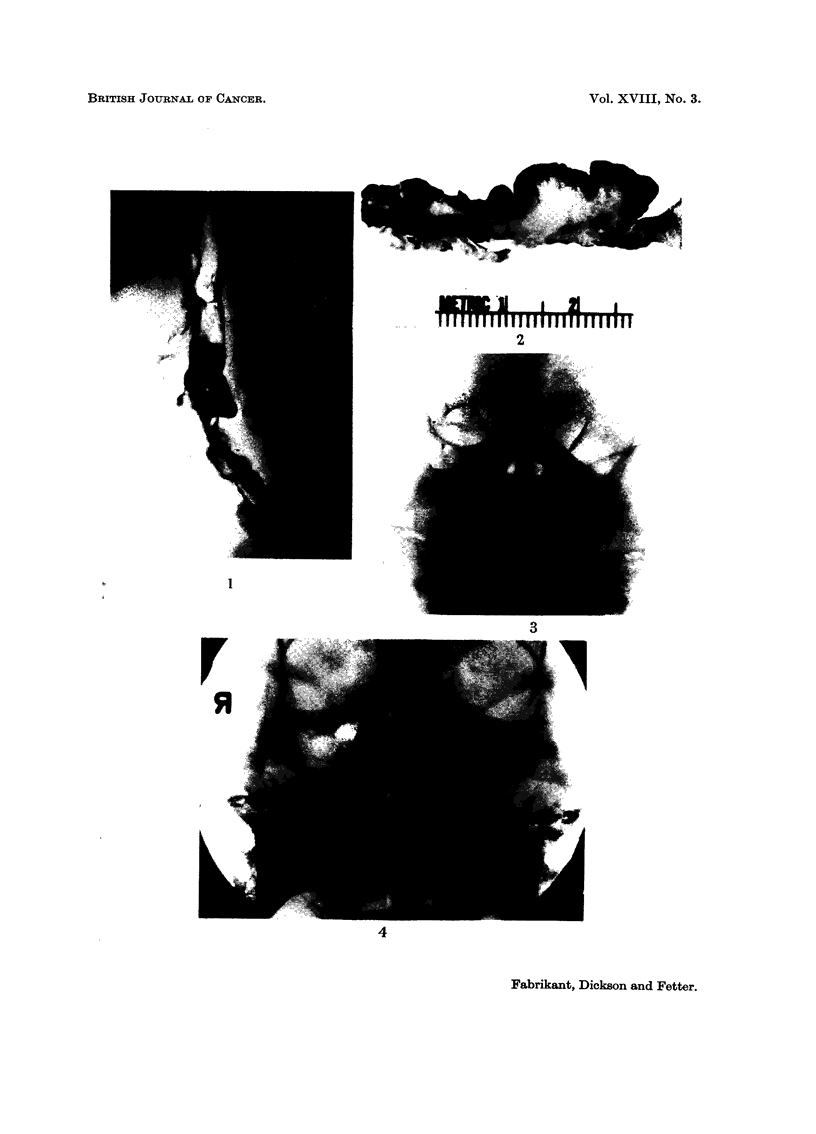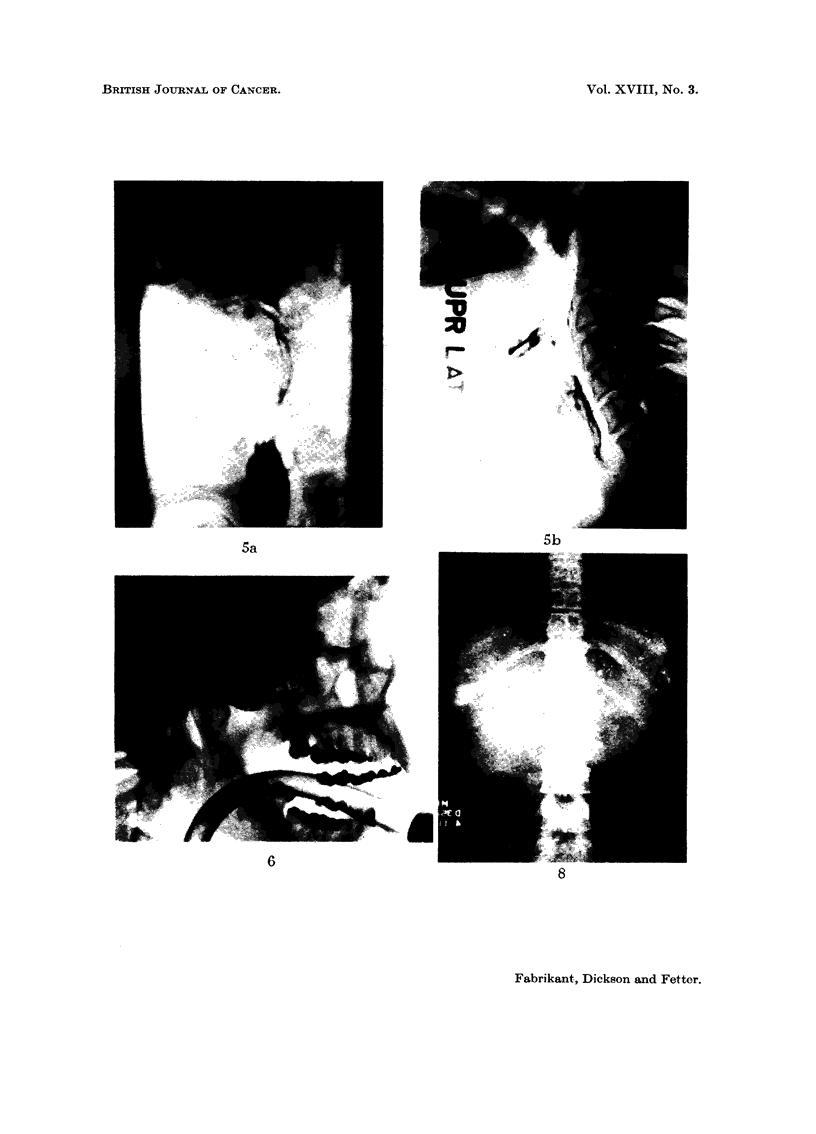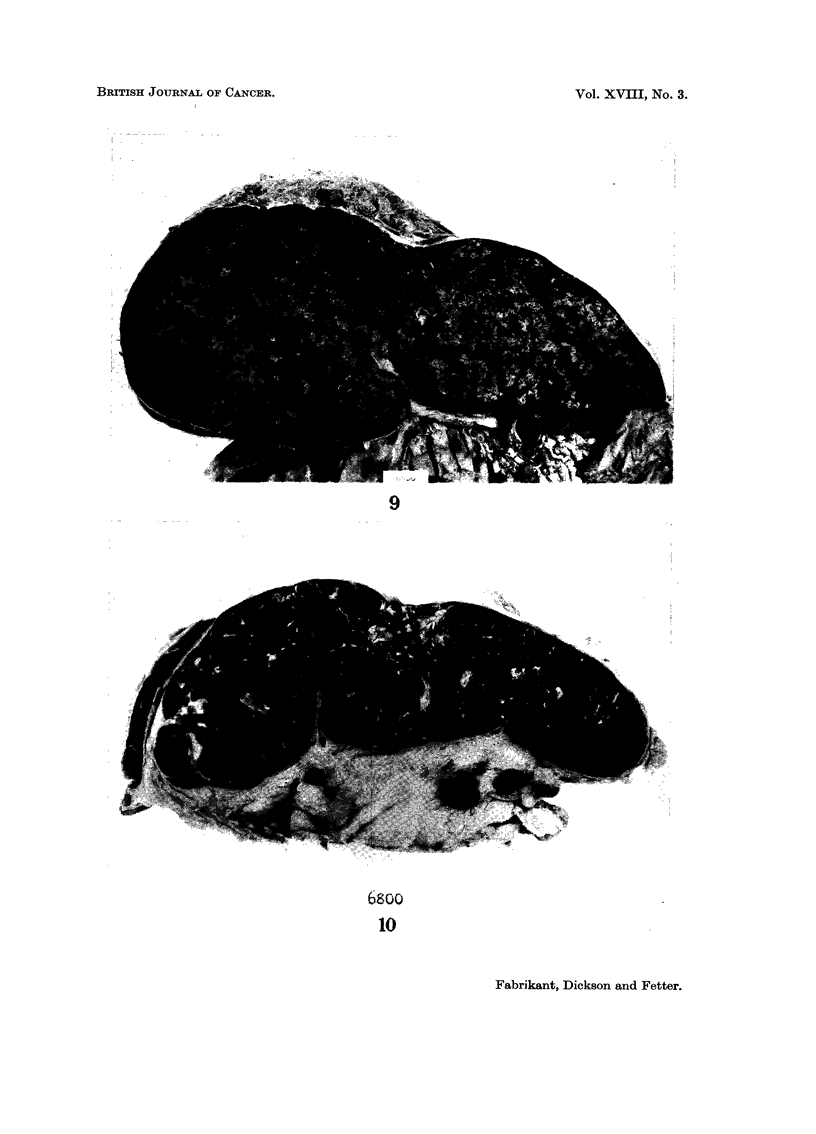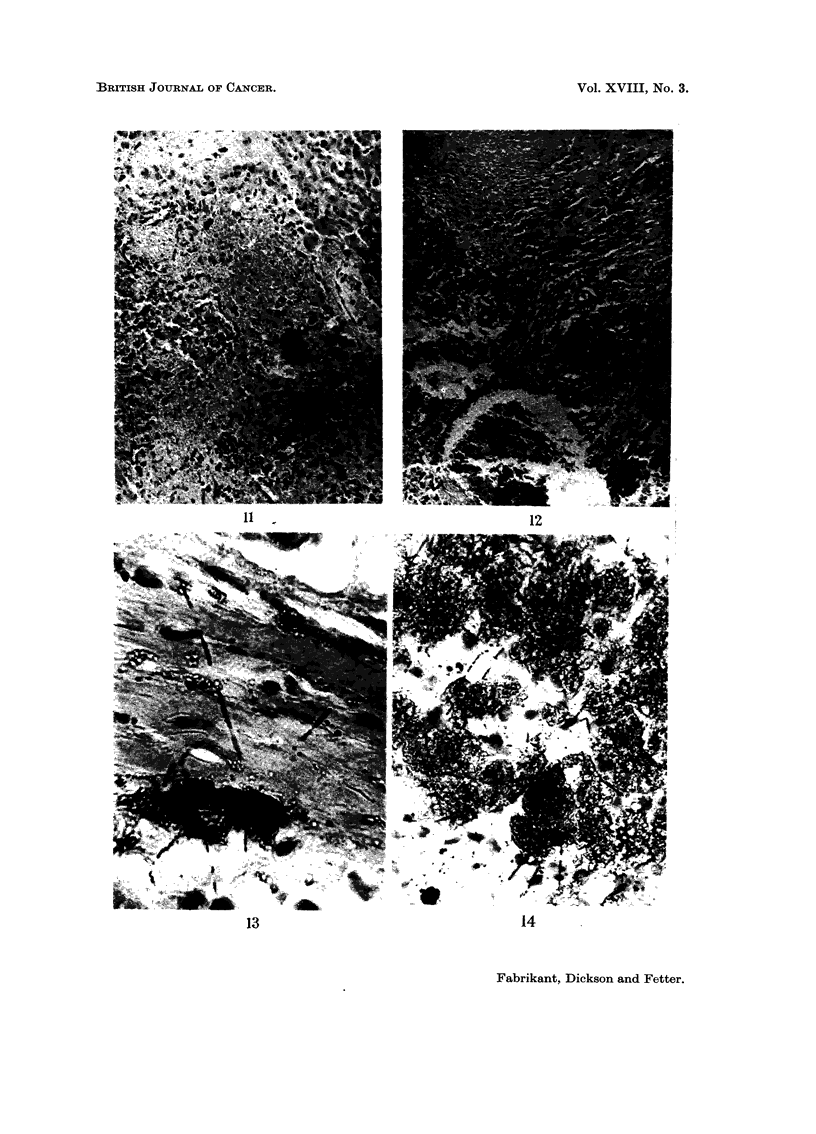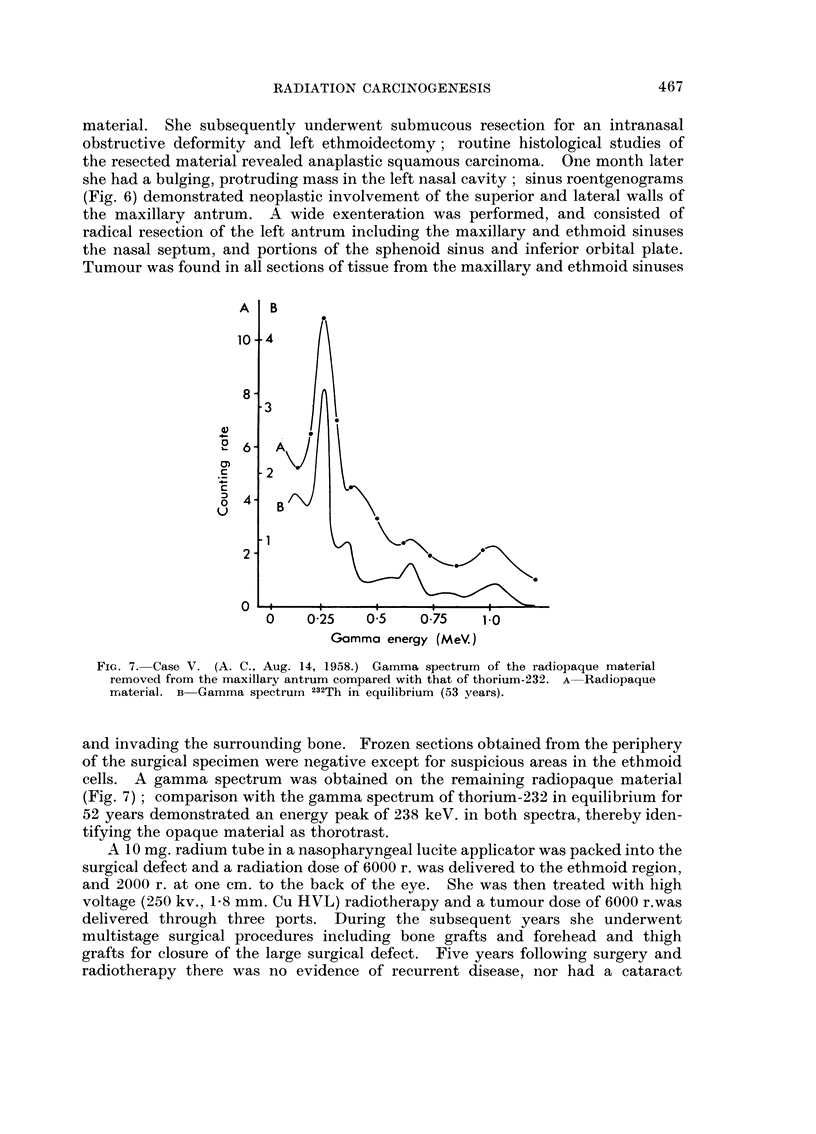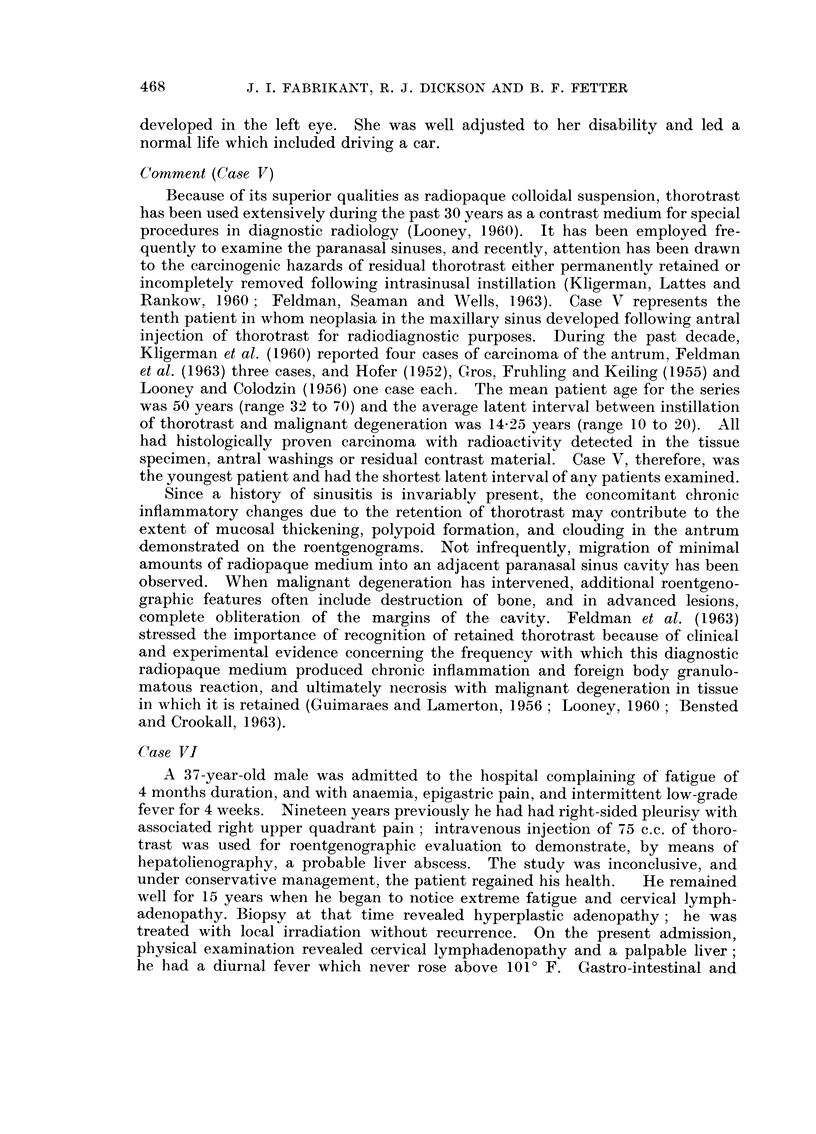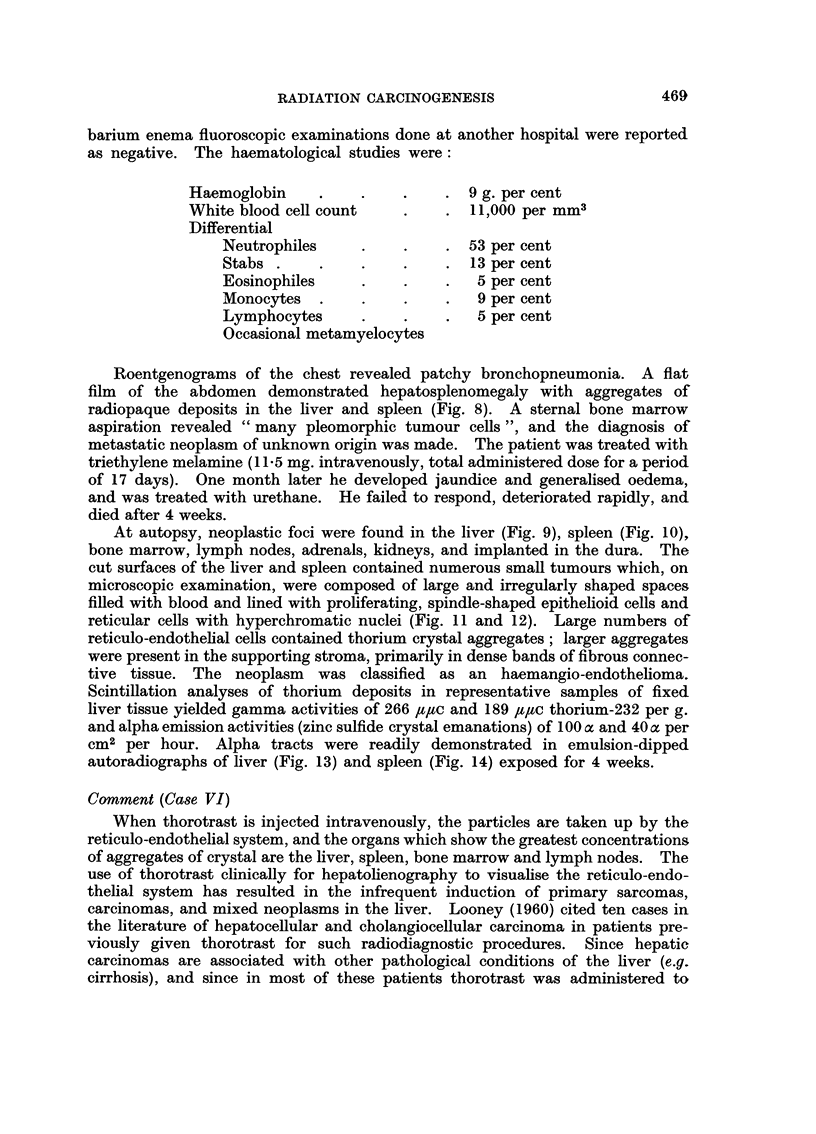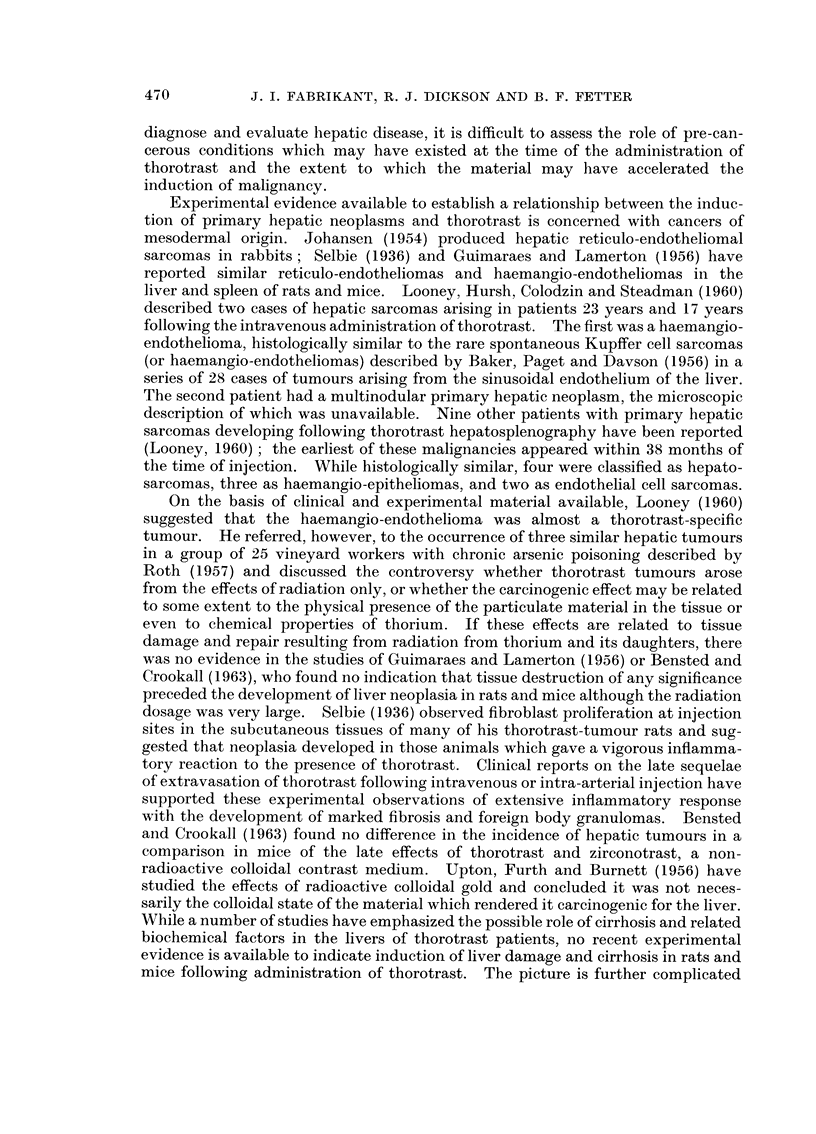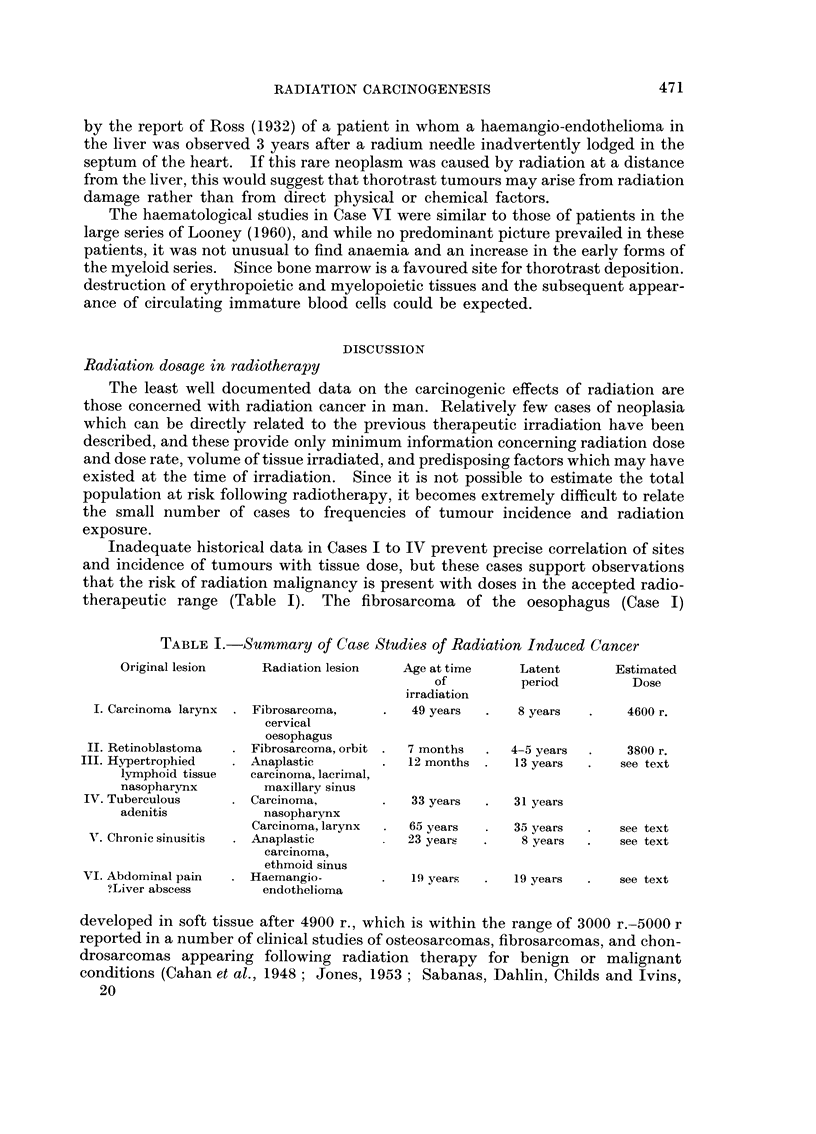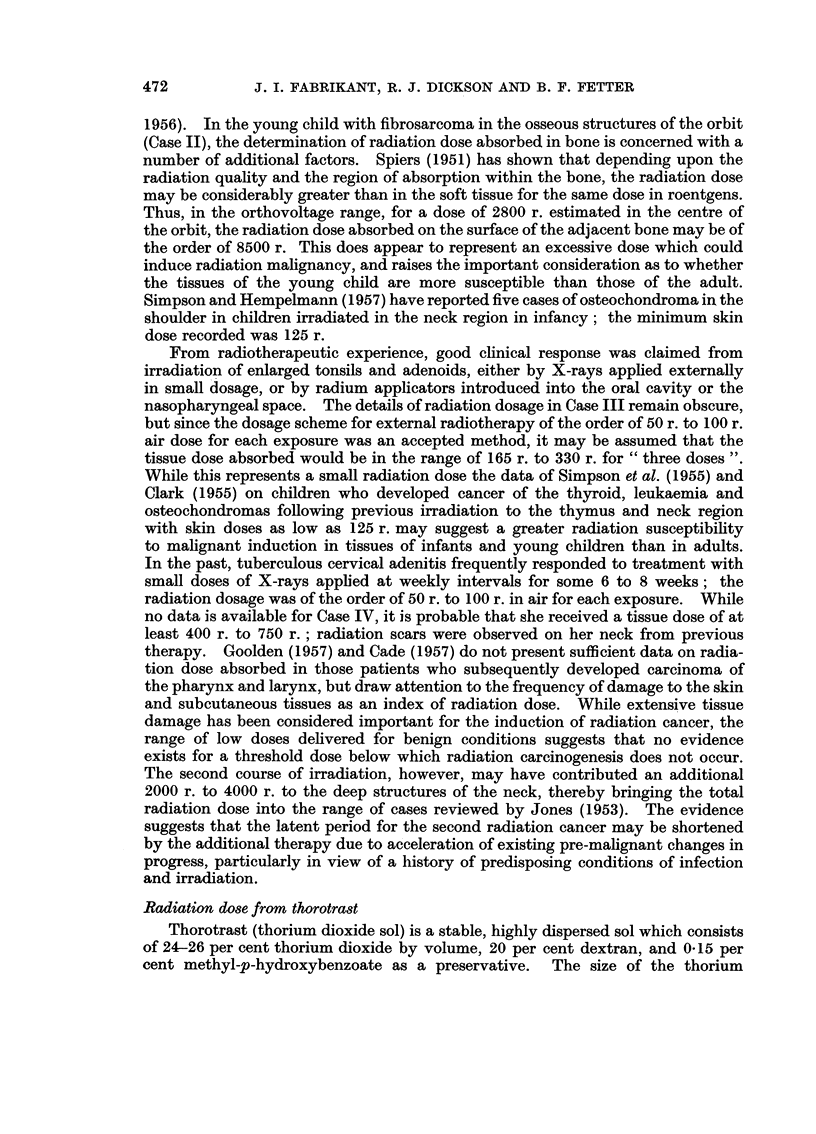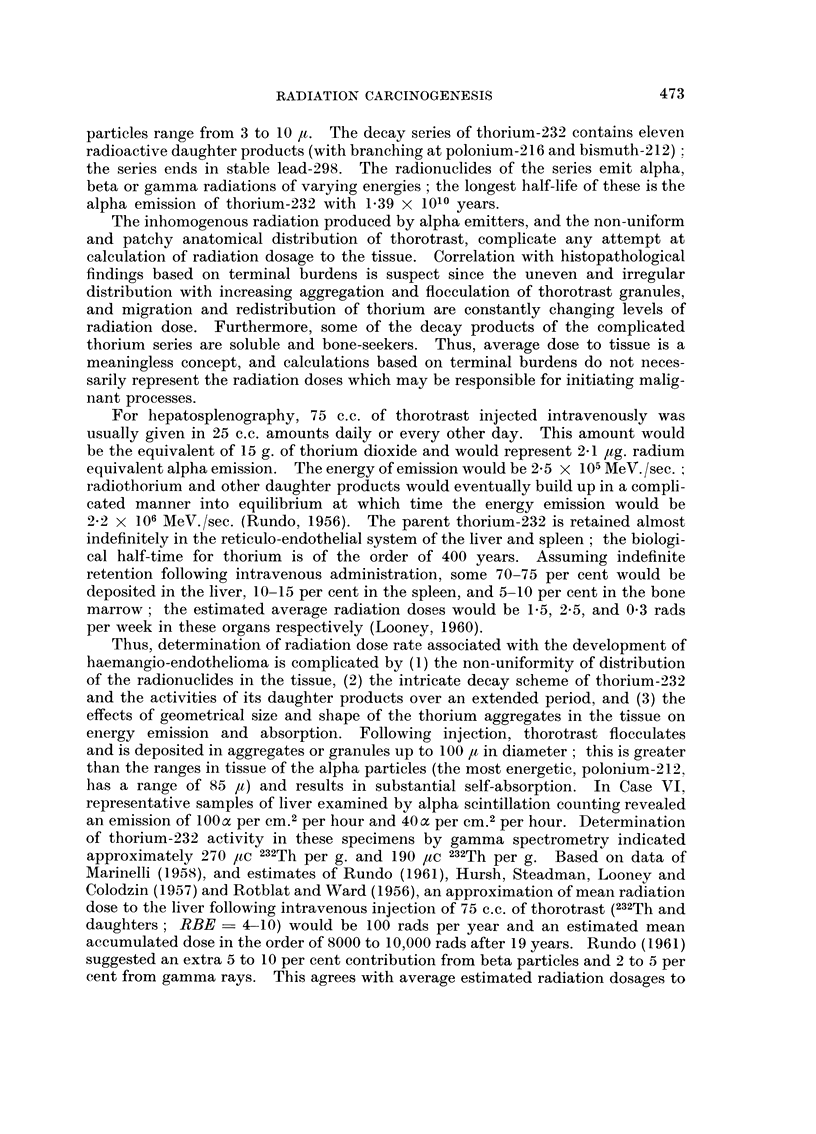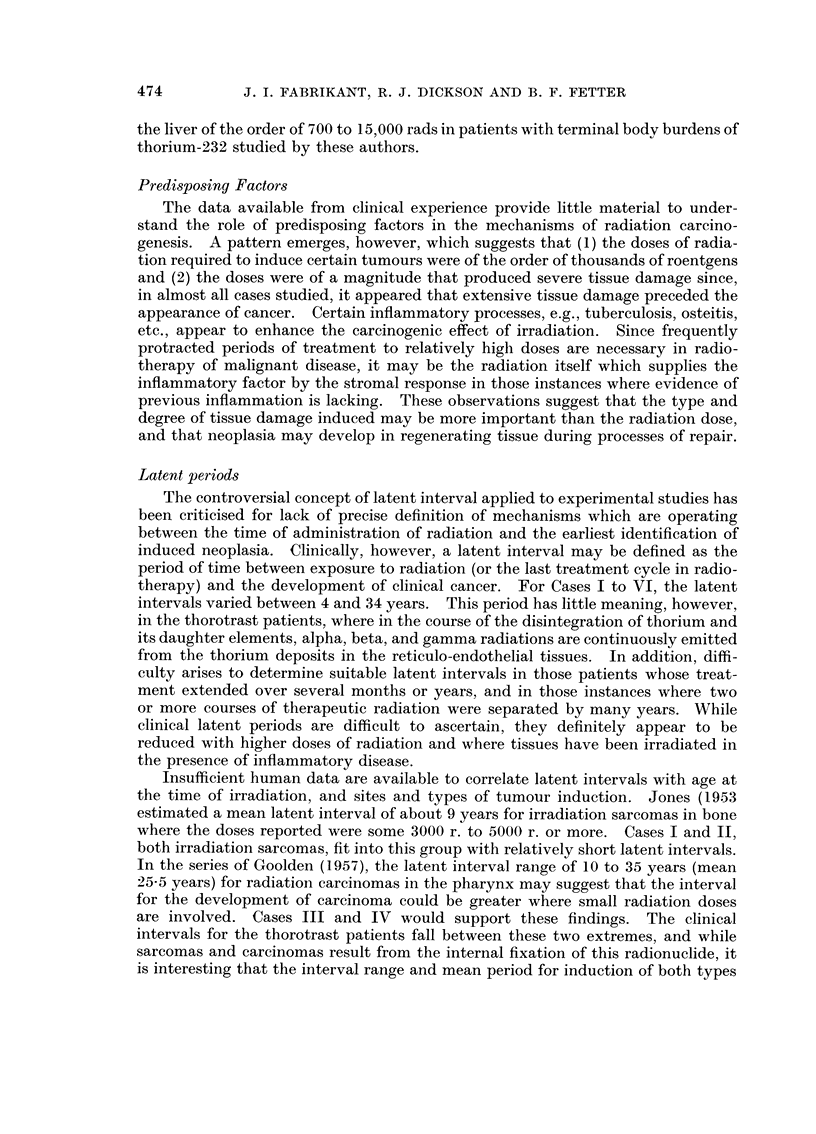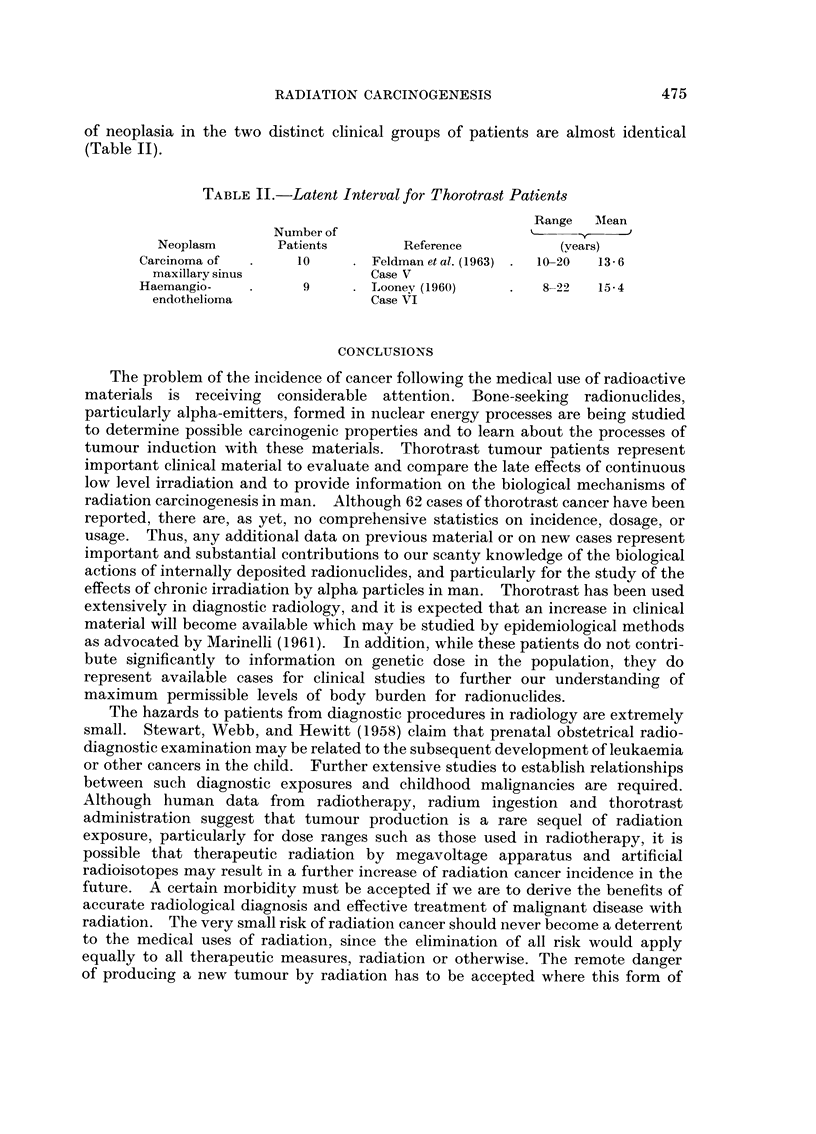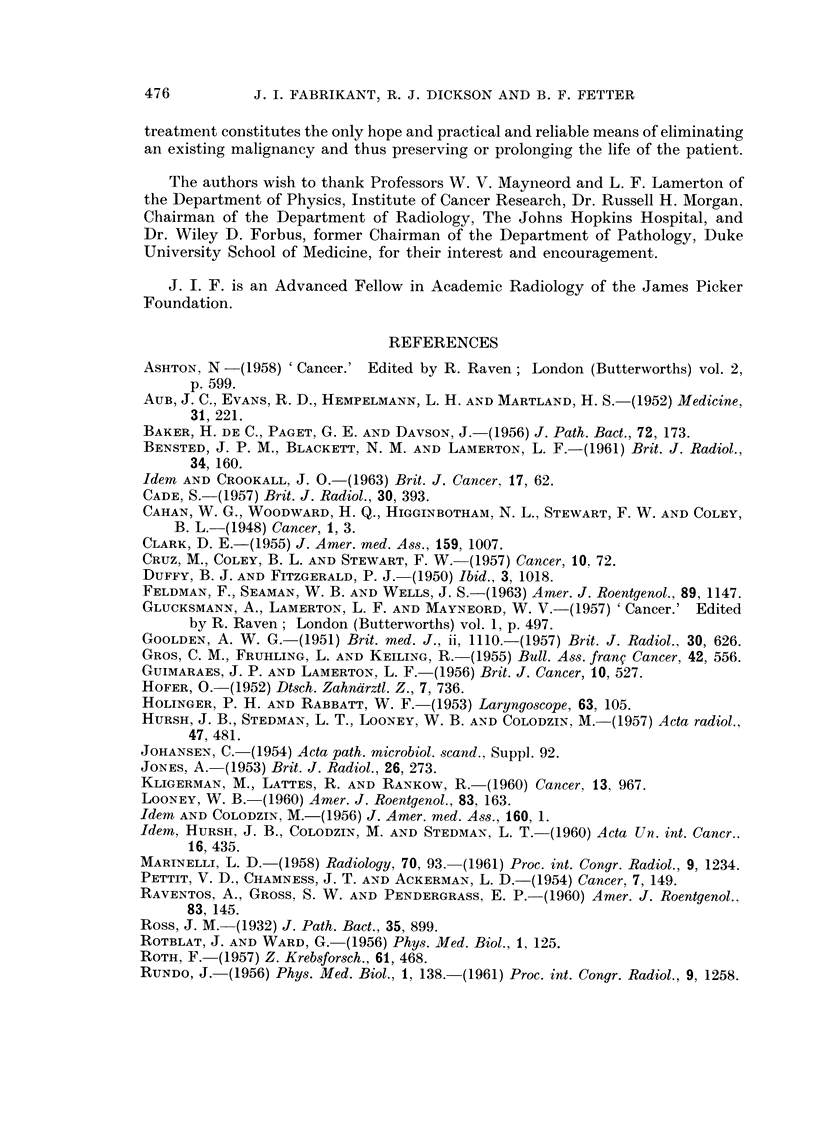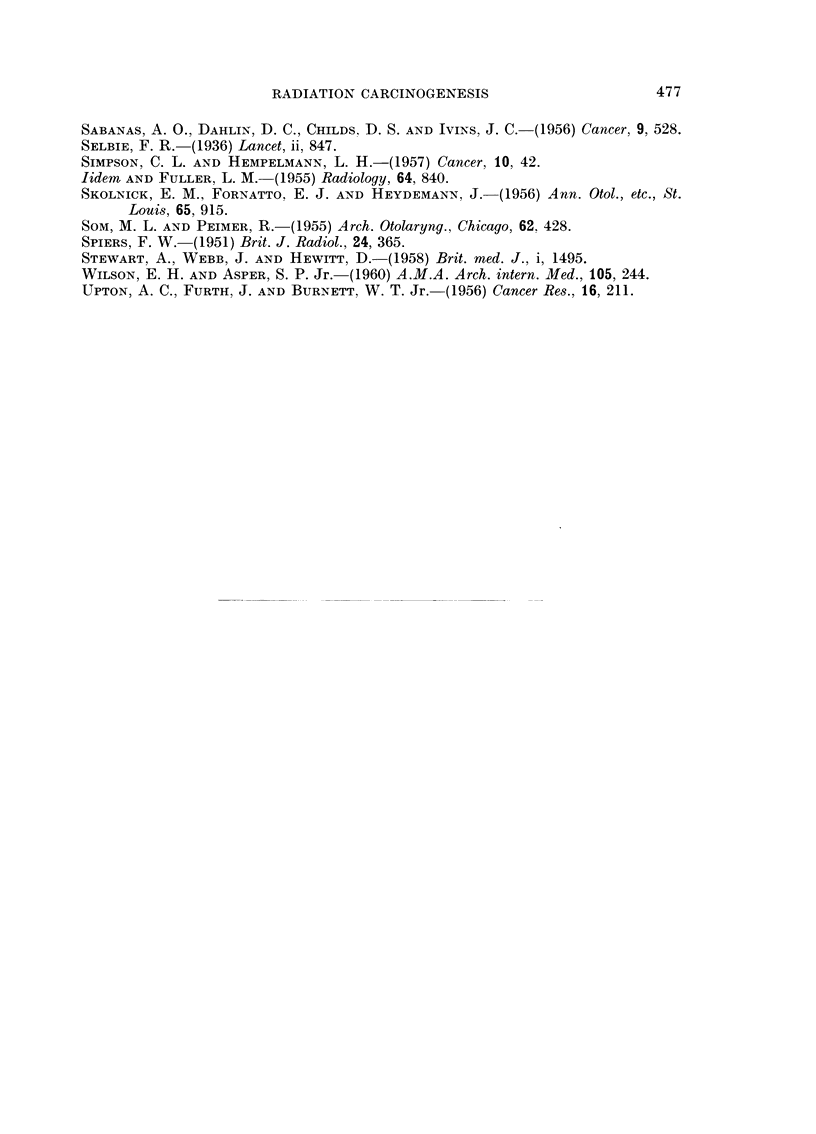# Mechanisms of Radiation Carcinogenesis at the Clinical Level

**DOI:** 10.1038/bjc.1964.52

**Published:** 1964-09

**Authors:** J. I. Fabrikant, R. J. Dickson, B. F. Fetter

## Abstract

**Images:**


### MECHANISMS OF RADIATION CARCINOGENESIS AT THE CLINICAL LEVEL

J. I. FABRIKANT*,[1] R. J. DICKSON[2] AND B. F. FETTER[3]

*From the [1]Department of Physics, Institute of Cancer Research, Belmont, Sutton, Surrey, [2]Radiotherapy Department, London Clinic, London, and [3]Department of Pathology, Duke University School of Medicine, Durham, North Carolina, U.S.A.*

Received for publication May 1, 1964

THE problem of malignancy as a complication of radiation has become a subject of major interest in recent years, primarily due to the increased use of radio-elements in industry, the widespread application of radiation techniques in medicine, and the concern with nuclear fall-out from weapons testing. Data on experimental carcinogenesis have emphasised cause and effect relationships and tumour incidence, but have provided little information on the antecedents of tumour induction related to radiation dose and dose rate, tissue response, and changes occurring between radiation exposure and the appearance of neoplasia. Even less information is available from the relatively small amount of human data obtained from clinical studies of radiation-induced cancer.

Data gathered from clinical reports, however, particularly on localised radiation, indicate that doses required for cancer induction may be of a magnitude to produce some form of observable tissue damage (Cade, 1957 ; Cahan, Woodward, Higginbotham, Stewart and Coley, 1948 ; Glucksmann, Lamerton and Mayneord, 1957). This may not necessarily be associated with the result of architectural or cellular disorientation, since radiotherapeutic experience suggests radiation cancer to be an unusual sequel. While certain malignancies appear to be causally related to preceding irradiation, it is probable that the incidence of cancer following irradiation may be higher than is apparent and that some tumours occur without evidence of demonstrable radiation tissue damage.

Malignancy has been recognised as a late complication of radiation exposure for over 50 years. A study of radiation-induced cancer in man during this interval reveals two periods of increased incidence. The first was due to occupational and industrial exposure and affected those whose work brought them into contact with radiation. The second period affected patients treated for benign or malignant diseases and reports of these cases have appeared in the medical literature with regularity during the past 25 years. It has become evident that both groups were exposed to excessive external or internally administered radiation during an era when the effects of ionizing radiations were poorly understood, dosimetry inaccurate, and protection inadequate. This report deals with the second group, and reviews the histories of six patients in whom cancer was induced as a consequence of radiation exposure made to treat a benign or malignant neoplasm or to establish a radiological diagnosis following internal administration of a radioactive contrast

* Present address: Department of Radiological Science, The Johns Hopkins University, 615 North Wolfe Street, Baltimore 5, Maryland, U.S.A.

material (thorotrast). These cases were studied and followed by the authors in the Radiotherapy Department of the Johns Hopkins Hospital. In order to assess the importance of the direct and indirect effects of radiation, relevant data on dose and dose rates, latent periods, extent of tissue damage, and types of tumours induced have been included whenever possible. These are discussed in terms of possible mechanisms of radiation carcinogenesis at the clinical level to provide additional information concerning the risks of diagnostic and therapeutic radiation exposure.

### CASE STUDIES

### *Case I*

A 49-year old male complained of intermittent hoarseness which had progressively worsened during the previous 7 months. Examination revealed a squamous cell carcinoma of the intrinsic larynx with involvement of the entire right vocal cord, the anterior commissure, and the anterior one-third of the left vocal cord. No lymph nodes were palpable. At total laryngectomy, it was found that tumour invaded the thyroid cartilage and extended into the pre-epiglottic space. Postoperatively, high voltage (250 kv., 1·8 mm. Cu HVL) radiotherapy was administered through two 6 cm. × 10 cm. lateral opposing ports ; however, the patient elected to return to his home city for completion of radiotherapy after 300 r. mid-line tissue dose had been delivered. The radiotherapist's report indicated that the patient received an additional 4600 r. tumour dose in over 4 weeks through similar lateral parallel opposing fields ; the estimated daily midline tissue dose was 162 r. The radiation quality is not known. On the completion of therapy, the patient experienced a " brisk " skin reaction moderate dysphagia, and soreness around the tracheostomy tube.

He did quite well following laryngectomy and radiation therapy until 8 years later when he complained of progressive dysphagia and loss of weight (11 pounds) for 3 months. He was unable to swallow food unless it was finely chewed or chopped, but he did not experience any pain. On examination, he appeared healthy and alert, breathed well through a healed tracheostomy, and spoke effectively with the aid of an electronic larynx. The skin of his neck was firm and inelastic with marked scarring and telangiectasia over the irradiated fields. A barium swallow fluoroscopic examination (Fig. 1) demonstrated multiple, nodular, polypoid irregularities involving the cervical oesophagus, extending up behind the pharynx, and displacing the trachea anteriorly. The cervical oesophagus was deviated to the right. The appearance was that of a retrotracheal and retropharyngeal mass, and considered a probable recurrent carcinoma with secondary invasion of the oesophagus. A hard, polypoid hypopharyngeal mass was observed at endoscopy ; however, a biopsy revealed no evidence of tumour and only chronic inflammatory tissue with extensive fibrosis which was compatible with marked radiation reaction.

The superior oesophagus was explored and when frozen section examination revealed polypoid sarcoma suggestive of leiomyosarcoma, the cervical oesophagus was resected and subsequent definitive procedures were to be based on the pathological findings. The lesion was a large polypoid fibrosarcoma, well confined to the oesophagus, with extensive dense fibrotic connective tissue and round cell infiltration ; the stigmata of chronic radiation reaction and the classical appearance of fusiform epithelioid sarcomatous cells suggested the tumour was radiationinduced. The lobulated mass arose diffusely from the mucosa over an area 5 cm. in diameter to fill the lumen (Fig. 2) and replaced the superficial muscularis propria and submucosa. The proximal oesophageal stump contained marked chronic inflammatory changes with fibrosis and foreign body reaction, but no tumour was present in the resected edge.

One week later an ileocolic transplant was performed ; the terminal ileum and ascending colon were brought up in a retrosternal position into the chest and neck and anastomosed to the proximal stump of the cervical oesophagus. Ileotransverse colostomy and gastrocolic anastomosis was done. His post-operative course was complicated by abdominal distension and intermittent fever of unknown origin which required exploratory laparotomy. Two days later, following a cerebrovascular accident, he suddenly became comatose, developed left hemiplegia, and died.

*Comment (Case I)*

This is the first patient with irradiation fibrosarcoma arising in the oesophagus following radiotherapy known to the authors. In the Manchester Series reviewed by Goolden (1957), one patient developed cancer in the oesophagus 35 years following irradiation for thyrotoxicosis ; the histological diagnosis was not recorded, but the author definitely excluded sarcoma. Previously, Goolden (1951) reported one patient with fibrosarcoma arising in the postcricoid region 30 years following irradiation for hyperthyroidism, and Holinger and Rabbatt (1953) described a fibrosarcoma arising from the aryepiglottic fold of the epipharynx 27 years after the patient had been irradiated for tuberculous lymphadenitis. Som and Peimer (1955) reported two cases of postcricoid carcinoma following irradiation for carcinoma of the larynx, but no cases are known where irradiation sarcoma has arisen following high voltage deep radiotherapy for a previous malignancy. Incomplete data make correlation between dose and type of cancer induced difficult to assess, but it is generally recognised that the sarcomas arise following higher tissue doses. The short interval period of 7 years in this patient agrees more with the mean latency of irradiation bone sarcoma (8·6 years) reported by Jones (1953) in a review series of 39 cases than with the interval of 25 to 30 years recorded by Goolden (1957) for 32 cases of irradiation carcinoma of the pharynx and oesophagus, and with that of the industrial epithelial tumours of known aetiology.

*Case II*

A 7-month-old baby girl developed unilateral exophthalmos squint, and a highly vascularised sclera of the left eye ; the right eye was normal. A large retinoblastoma (endophytum type) was identified in the posterior chamber arising from the infero-posterior portion of the retina. Roentgenograms revealed no involvement of the osseous structures of the orbit, nor was there enlargement of the optic foramen. The eye was enucleated, and a wide exenteration of the orbital contents was performed. Pathological examination revealed the classical appearance of true rosettes of carrot-shaped cells with scanty cytoplasm and hyperchromatic nuclei ; tumour extended through the outer coats of the eye, but there was no invasion of the optic nerve or peri-orbital fat. Postoperative high voltage radiotherapy (250 kv., 1·8 mm. Cu HVL) was administered ; the radiation dose to the posterior wall of the orbit was estimated to be 2800 r. delivered through two fields (antero-posterior and lateral) angled at 85 degrees to each other during a period of 4 weeks. The child did well, and moderate skin reaction was noted in the treated areas.

There was no evidence of recurrent tumour $3\frac{1}{2}$ years later, but while the child was being measured for a prosthesis, she complained of tenderness along the lateral wall of the left orbit. Radiographs revealed early changes of radiation osteitis but no overt bone destruction. Eighteen months later (5 years following surgery) a large mass had become palpable and the child complained of continuous pain and marked tenderness. X-ray examination demonstrated progression of the osteitis previously noted, but now there were irregular osteolytic areas of destruction in the lateral orbital wall, most prominent in the left frontal bone (Fig. 3). Biopsy of the mass was reported as fibrosarcoma. A wide excision was carried out and revealed a dense spindle cell fibrosarcoma with occasional giant cells, but no evidence of new bone formation. Chronic inflammatory changes of radiation osteitis were identified in the adjacent bone. The child survived for $2\frac{1}{2}$ years and died with generalised sarcomatous metastases. There was no recurrence of the retinoblastoma.

*Comment* (*Case II*)

The induction of spindle cell fibrosarcoma following external irradiation has been frequently described (Cahan, *et al.*, 1948 ; Cruz, Coley and Stewart, 1957 ; Jones, 1953) ; however, few reports are known to the authors of the development of fibrosarcoma following radiation injury to bone in a young child and with a brief latent interval of only 4 years. The criteria for post-irradiation sarcoma established by Cahan *et al.* (1948) were fulfilled, namely (1) roentgenographic evidence of normal bone before irradiation, (2) development of sarcoma in tissue included in the radiotherapeutic beam, (3) a symptom-free latent interval and (4) histological proof of sarcoma in previously normal bone and adjacent tissues. In the cases of irradiation osteosarcoma reviewed by Jones (1953), the latent periods varied from 3 to 22 years with a mean interval of 8·6 years ; the age of the patients ranged from 9 to 62 years. All occurred following external irradiation, but in none could any phase during the latent period be recognised as osteoradionecrosis, although in 13 patients the tumour arose in previously normal bone. An additional 14 cases of radiation sarcoma of the skull have been reported (Cruz *et al.*, 1957 ; Raventos, Gross and Pendergrass, 1960 ; Skolnik, Fornatto, and Heydemann, 1956), and in none of these was radiation injury to bone observed to precede malignant degeneration, although Cruz *et al.* (1957) referred to two patients in whom radiographic evidence suggested radiation osteitis was antecedent to the induction of sarcoma. Petit, Chamness and Ackerman (1954) discussed fibrosarcoma in the deep connective tissues following external radiation therapy in three patients, but none was related to radiation injury to bone. Raventos *et al.* (1960) described the appearance of fibrosarcoma in the osseous calvarium or in the overlying galea aponeurotica in a patient irradiated for a pituitary tumour 17 years before, and in whom roentgenographic changes of radiation osteitis had been present for 7 years. However, there was no histological evidence of radiation osteitis or radionecrosis in the tissue fragments examined, and it was suggested that the osteolytic lesions observed roentgenographically represented resorption, rather than necrosis, following radiation injury to the calvarium. Since Case II had histological evidence of radiation osteitis, the history of this child was similar to those patients of Aub, Evans, Hempelmann, and Martland (1952), Looney (1960) and others in whom sarcomas developed in regions of radiation osteitis following the internal deposition of radioactive nuclides (radium and/or mesothorium).

The clinical features of malignant change most frequently observed in the patients of Cahan *et al.* (1948), namely, unremitting and progressively increasing pain and swelling in an area which had been previously irradiated, were observed in this patient. Although the clinical diagnosis of radiation osteitis was verified it does not appear that the condition was followed radiographically to determine interval improvement or deterioration. While sarcomatous degeneration could not be confirmed without biopsy, malignancy could have been suspected from the early osteolytic changes. Pathological experience indicates that a wide variety of characteristic histological features of these sarcomas exist ; pleomorphic spindle cell sarcomas may arise in intimate relation to osseous structures, but often new bone is poorly formed or actually deficient. Thus, the roentgenographic and microscopic features in Case II are somewhat similar to those described in the patient of Raventos *et al.* (1960) ; the additional histological evidence of characteristic radiation osteitis and necrosis preceding the development of fibrosarcoma in this child was an important additional finding.

*Case III*

At 12 months of age, this boy received " X-ray treatments " to the tonsils for tonsillitis and for hypertrophied lymphoid tissue in the nasopharynx. The method of application, radiation quality and dosage are not known, except that he was given "three doses ". At 14 years, he complained of spontaneous nose bleeds and nasal obstruction on the left. Following mumps meningitis one year later, left exophthalmos persisted without diplopia or headache, but the child experienced occasional bilateral tinnitus. Roentgenograms (Fig. 4) demonstrated complete clouding of the left maxillary antrum with destruction of the left lateral sinus wall and the lateral and inferior orbital walls by a malignant process. Destruction and opacification extended into the ethmoid and sphenoid sinuses. He underwent complete exenteration of the orbit and adjacent maxillary and ethmoid sinuses to include the lateral wall of the sphenoid sinus. The tumour extended into the nasopharynx and posteriorly into the retro-orbital fat, along the optic nerve and into the foramen, into the surrounding bone, and involved the cribriform plate. Histological examination revealed a highly anaplastic adenocarcinoma (adenoid cystic type) of lacrimal duct origin with an extensive fibrovascular connective tissue stroma, and chronic inflammatory changes with round cell infiltration and foreign body reaction. Tumour was present in the posterior margin of the surgical specimen. A mold containing two 25 mg. radium capsules in dental wax was placed in the surgical defect ; this delivered 7000 r. at 0·5 cm. within the residual tumour. External high voltage radiotherapy (250 kv., 1·8 mm. Cu HVL) supplemented the radium therapy ; 3000 r. tumour dose was administered in 6 weeks through two angled ports with shielding of the right eye.

Five months later he complained of unremitting pain in the left temporal and occipital regions. He was unable to open his mouth due to ankylosis of the left temporomandibular joint secondary to marked osteoradionecrosis and osteomyelitis of the ascending ramus of the left mandible. This required further extensive surgical care involving osteotomy with excision of the ascending ramus and condyle of the left mandible and débridement of an orbital sequestrum; there was marked trismus of the left masseter and pterygoid muscles. The child had a stormy postoperative period complicated by recalcitrant cellulitis and abscess formation in the left cheek, and by a moderate depressive reaction. Three years later, no recurrent neoplasm was observed.

*Comment (Case III)*

Tumours of the lacrimal sac and duct are uncommon; only 124 cases have been reported (reviewed by Ashton, 1958). While these appear most frequently in the fifth decade of life, a case was recorded in a 13-year-old child. Of the malignant tumours, epitheliomas are the most common. These are closely related histologically and developmentally to similar carcinomas arising in the nasal and paranasal cavities since the lining epithelium of the sac and duct and the respiratory epithelium develop along similar embryologic lines. The tumour usually follows episodes of chronic inflammation, may undergo widespread proliferative changes in the epithelium, and show invasive tendencies at an early stage. The more malignant lesions are poorly differentiated and consist of irregular masses of hyperchromatic, pleomorphic or cylindrical cells; cyst formation is common, and proliferation of malignant cells may occur within the cysts in a manner similar to intraductal carcinoma.

None of the cases reported suggest a previous history of radiation; however, since no indication that a specific inquiry about radiation was made, this information may be frequently absent from chart histories taken before the relationship between radiation and head and neck tumours was established. Goolden (1957) described a number of types of irradiation cancers arising in the upper air passages; none of the 32 cases of pharyngeal cancer, however, arose in the nasopharynx, paranasal sinuses, or lacrimal structures. A number of studies (Duffy and Fitzgerald, 1950; Simpson, Hempelmann and Fuller, 1955; Wilson and Asper, 1960) report a high incidence of thyroid tumors in children and adolescents previously irradiated in the neck and thoracic regions, most frequently for thymic or tonsillar enlargement and hypertrophied lymphoid tissue in the nasopharynx; leukemias and osteochondromas of the shoulder were the only other neoplasms observed in the irradiated groups with significant frequency when compared with control siblings. These findings raised the very important additional consideration whether the infant or young child is more susceptible than the adult to radiation-induced malignancy.

*Case IV*

In 1919, this 71-year-old female had irradiation to the left cervical lymph nodes for tuberculous lymphadenitis. She subsequently developed marked radiation scars in the overlying skin of her neck, but with only minimal subcutaneous induration. In 1953 she developed a large carcinoma in the left nasopharynx at the eustachian tube orifice which was treated as follows. A radium mold placed in the nasopharynx and against the eustachian tube orifice delivered 5750 r. at 0·5 cm.; high voltage (200 kv.) external radiotherapy to the left maxillary antrum delivered 4000 r. through 4 cm. × 4 cm. left lateral and right anterior maxillary ports, and through an intra-oral cone 1400 r. air dose. The radiation quality is not known.

Four years later she presented with hoarseness and progressive dysphagia of 3 months' duration. There was no recurrence of her nasopharyngeal carcinoma and her general physical examination was unremarkable except for a Grade II apical systolic murmur with a history of rheumatic heart disease, mitral insufficiency and aortic stenosis. On fluoroscopic examination a barium meal demonstrated her difficulty to initiate the swallowing mechanism and revealed the extent to which an irregular tumour mass involved the hypopharynx and the oesophageal lumen (Fig. 5). At laryngoscopy, an extensive carcinoma involving both arytenoids, the epiglottis, cricopharyngeus, left pyriform sinus and the entrance to the oesophagus was observed ; the true vocal cords moved slightly. Biopsies, positive for squamous cell carcinoma, were obtained from the left arytenoid, the left cricopharyngeus, and the left side of the petiole of the epiglottis. Bronchoscopic examination revealed slight generalised irritation of the endobronchial tree, but no purulent material or other exudate.

Treatment consisted of rotational high voltage (250 kv. 1·8 mm. Cu HVL) radiotherapy with shielding of the radiation scars on her neck. A total dose of 5000 r. in 17 treatments was delivered to the midline of the hypopharynx and larynx. She developed a marked skin reaction with desquamation primarily on the left side, while her right side showed only minimal skin changes. Because of the soreness and mucositis in her mouth, radiotherapy was discontinued. At the conclusion of treatment, she still experienced dysphagia but there was improvement in her voice. Examination of the larynx at that time revealed some oedema of the arytenoids, but no evidence of ulceration ; the size of the mass had diminished considerably. Three months later her voice and ability to swallow were much improved, although she had now developed a deep ulcer on the left arytenoid which was considered as radiation necrosis. Six months following therapy, she complained of progressive pain in her throat radiating to her left ear ; the oedema and ulceration of the treated structures had worsened and residual neoplasia was observed. Her condition deteriorated and she died within one year with extensive recurrence of her disease.

*Comment* (*Case IV*)

In the series reviewed by Goolden (1957) all of the 42 patients, in whom radiation cancer developed in the deep tissues of the neck, had been treated previously with irradiation for a benign condition, primarily tuberculous lymphadenitis and thyrotoxicosis. In none of the 32 patients in this group with radiation cancer in the pharynx did carcinoma arise in the nasopharynx ; in 31 it was confined to the epipharynx, and in one to the oesophagus. In Case IV, the latent interval of 34 years agrees well with the range of 10 to 35 years in that series, but it is somewhat greater than the mean interval of 25·5 years. While the patient's second carcinoma involving the extrinsic larynx and cervical oesophagus may have been causally related to her irradiation 38 years before, it is probable that its induction was accelerated by the second course of deep radiotherapy 4 years previously. Clinical experience is lacking on the carcinogenic effects of chronic or repeated doses of irradiation ; however, experimental evidence has demonstrated convincingly the enhanced carcinogenicity of chronic or repeated irradiation, both from external X-ray and gamma radiation and from internally administered radionuclides (Bensted, Blackett and Lamerton, 1961) which is manifested by an increased percentage and earlier onset of tumours.

There is an apparent increased frequency with which tumours of the pharynx, induced following irradiation for tuberculous adenitis, have their primary site of origin in the laryngopharynx ; the majority of these are confined to the epiglottis, aryepiglottic folds and lateral pharyngeal walls. These are relatively uncommon sites for the distribution of cancer of the pharynx in woman where lesions arising in the oropharynx and epipharynx are three to four times less frequent in females than in males. Jones (1953), Goolden (1957), Cade (1957), and others stress the clinical importance of overlying skin changes following heavy irradiation since chronic radiodermatitis, fibrosis, telangiectasia, and subcutaneous induration are often reliable indices of dosage in patients whose histories lack adequate physical estimates of radiation dose. Repeated irradiation would accelerate the latent period, and although frank neoplasia may not be present until the latent period is completed, following this the development of a second malignancy may be extremely rapid.

*Case V*

A 31-year-old female complained of chronic sinusitis and asthma since childhood. In 1950, a radiopaque liquid was instilled in the left maxillary antrum for diagnostic examination ; none of the fluid was removed. Eight years later she presented with an acute exacerbation of her sinusitis ; roentgenograms at that time revealed residual contrast medium in the antrum. She was treated with antibiotics and irrigations and drainage which removed most of the remaining

---

EXPLANATION OF PLATES

FIG. 1.—Case I. Barium swallow roentgenogram demonstrating a multinodular polypoid tumour involving the cervical oesophagus 8 years following irradiation.

FIG. 2.—Case I. Polypoid fibrosarcoma arising diffusely from the oesophageal mucosa.

FIG. 3.—Case II. Reontgenographic evidence of irregular osteolytic destruction of the lateral wall of the left orbit 5 years following irradiation.

FIG. 4.—Case III. Clouding of the left maxillary antrum and the adjacent paranasal sinuses and irregular destruction of the infero-medial wall of the orbit 13 years following irradiation.

FIG. 5.—Case IV. Barium swallow roentgenograms demonstrating an irregular tumour mass involving the hypopharynx and oesophageal lumen 38 years following the first, and 4 years following the second course of irradiation. A. Anteroposterior, B. Lateral.

FIG. 6.—Case V. Neoplasm involving the left maxillary sinus and adjacent structures 8 years following antral instillation of thorotrast.

FIG. 8.—Case VI. Roentgenogram of the abdomen demonstrating hepatosplenomegaly with aggregates of radiopaque deposits in the liver and spleen 19 years following thorotrast hepatolienography.

FIG. 9.—Case VI. Haemangio-endothelioma in the liver 19 years following thorotrast hepatolienography.

FIG. 10.—Case VI. Haemangio-endothelioma in the spleen 19 years following thorotrast hepatolienography.

FIG. 11.—Case VI. Haemangio-endothelioma in the liver ; thorotrast crystals are present in the reticulo-endothelial cells. × 135.

FIG. 12.—Case VI. Haemangio-endothelioma in the spleen ; thorotrast crystals are present in the reticulo-endothelial cells. × 135.

FIG. 13.—Case VI. Autoradiography demonstrating alpha tracks from thorotrast crystals in the liver. ×270 oil.

FIG. 14.—Case VI. Autoradiography demonstrating alpha tracks from thorotrast crystals in the spleen. ×270 oil.

1

2

3

4

5a

5b

6

8

9

10

Fabrikant, Dickson and Fetter.

11

12

13

14

Fabrikant, Dickson and Fetter.

material. She subsequently underwent submucous resection for an intranasal obstructive deformity and left ethmoidectomy ; routine histological studies of the resected material revealed anaplastic squamous carcinoma. One month later she had a bulging, protruding mass in the left nasal cavity ; sinus roentgenograms (Fig. 6) demonstrated neoplastic involvement of the superior and lateral walls of the maxillary antrum. A wide exenteration was performed, and consisted of radical resection of the left antrum including the maxillary and ethmoid sinuses the nasal septum, and portions of the sphenoid sinus and inferior orbital plate. Tumour was found in all sections of tissue from the maxillary and ethmoid sinuses

Fig. 7.—Case V. (A. C., Aug. 14, 1958.) Gamma spectrum of the radiopaque material removed from the maxillary antrum compared with that of thorium-232. A—Radiopaque material. B—Gamma spectrum $^{232}$Th in equilibrium (53 years).

and invading the surrounding bone. Frozen sections obtained from the periphery of the surgical specimen were negative except for suspicious areas in the ethmoid cells. A gamma spectrum was obtained on the remaining radiopaque material (Fig. 7) ; comparison with the gamma spectrum of thorium-232 in equilibrium for 52 years demonstrated an energy peak of 238 keV. in both spectra, thereby identifying the opaque material as thorotrast.

A 10 mg. radium tube in a nasopharyngeal lucite applicator was packed into the surgical defect and a radiation dose of 6000 r. was delivered to the ethmoid region, and 2000 r. at one cm. to the back of the eye. She was then treated with high voltage (250 kv., 1·8 mm. Cu HVL) radiotherapy and a tumour dose of 6000 r.was delivered through three ports. During the subsequent years she underwent multistage surgical procedures including bone grafts and forehead and thigh grafts for closure of the large surgical defect. Five years following surgery and radiotherapy there was no evidence of recurrent disease, nor had a cataract developed in the left eye. She was well adjusted to her disability and led a normal life which included driving a car.

*Comment* (*Case V*)

Because of its superior qualities as radiopaque colloidal suspension, thorotrast has been used extensively during the past 30 years as a contrast medium for special procedures in diagnostic radiology (Looney, 1960). It has been employed frequently to examine the paranasal sinuses, and recently, attention has been drawn to the carcinogenic hazards of residual thorotrast either permanently retained or incompletely removed following intrasinusal instillation (Kligerman, Lattes and Rankow, 1960; Feldman, Seaman and Wells, 1963). Case V represents the tenth patient in whom neoplasia in the maxillary sinus developed following antral injection of thorotrast for radiodiagnostic purposes. During the past decade, Kligerman *et al.* (1960) reported four cases of carcinoma of the antrum, Feldman *et al.* (1963) three cases, and Hofer (1952), Gros, Fruhling and Keiling (1955) and Looney and Colodzin (1956) one case each. The mean patient age for the series was 50 years (range 32 to 70) and the average latent interval between instillation of thorotrast and malignant degeneration was 14·25 years (range 10 to 20). All had histologically proven carcinoma with radioactivity detected in the tissue specimen, antral washings or residual contrast material. Case V, therefore, was the youngest patient and had the shortest latent interval of any patients examined.

Since a history of sinusitis is invariably present, the concomitant chronic inflammatory changes due to the retention of thorotrast may contribute to the extent of mucosal thickening, polypoid formation, and clouding in the antrum demonstrated on the roentgenograms. Not infrequently, migration of minimal amounts of radiopaque medium into an adjacent paranasal sinus cavity has been observed. When malignant degeneration has intervened, additional roentgenographic features often include destruction of bone, and in advanced lesions, complete obliteration of the margins of the cavity. Feldman *et al.* (1963) stressed the importance of recognition of retained thorotrast because of clinical and experimental evidence concerning the frequency with which this diagnostic radiopaque medium produced chronic inflammation and foreign body granulomatous reaction, and ultimately necrosis with malignant degeneration in tissue in which it is retained (Guimaraes and Lamerton, 1956; Looney, 1960; Bensted and Crookall, 1963).

*Case VI*

A 37-year-old male was admitted to the hospital complaining of fatigue of 4 months duration, and with anaemia, epigastric pain, and intermittent low-grade fever for 4 weeks. Nineteen years previously he had had right-sided pleurisy with associated right upper quadrant pain; intravenous injection of 75 c.c. of thorotrast was used for roentgenographic evaluation to demonstrate, by means of hepatolienography, a probable liver abscess. The study was inconclusive, and under conservative management, the patient regained his health. He remained well for 15 years when he began to notice extreme fatigue and cervical lymphadenopathy. Biopsy at that time revealed hyperplastic adenopathy; he was treated with local irradiation without recurrence. On the present admission, physical examination revealed cervical lymphadenopathy and a palpable liver; he had a diurnal fever which never rose above 101° F. Gastro-intestinal and barium enema fluoroscopic examinations done at another hospital were reported as negative. The haematological studies were :

| | |
|---|---|
| Haemoglobin | 9 g. per cent |
| White blood cell count | 11,000 per $mm^3$ |
| Differential | |
| Neutrophiles | 53 per cent |
| Stabs | 13 per cent |
| Eosinophiles | 5 per cent |
| Monocytes | 9 per cent |
| Lymphocytes | 5 per cent |
| Occasional metamyelocytes | |

Roentgenograms of the chest revealed patchy bronchopneumonia. A flat film of the abdomen demonstrated hepatosplenomegaly with aggregates of radiopaque deposits in the liver and spleen (Fig. 8). A sternal bone marrow aspiration revealed " many pleomorphic tumour cells ", and the diagnosis of metastatic neoplasm of unknown origin was made. The patient was treated with triethylene melamine (11·5 mg. intravenously, total administered dose for a period of 17 days). One month later he developed jaundice and generalised oedema, and was treated with urethane. He failed to respond, deteriorated rapidly, and died after 4 weeks.

At autopsy, neoplastic foci were found in the liver (Fig. 9), spleen (Fig. 10), bone marrow, lymph nodes, adrenals, kidneys, and implanted in the dura. The cut surfaces of the liver and spleen contained numerous small tumours which, on microscopic examination, were composed of large and irregularly shaped spaces filled with blood and lined with proliferating, spindle-shaped epithelioid cells and reticular cells with hyperchromatic nuclei (Fig. 11 and 12). Large numbers of reticulo-endothelial cells contained thorium crystal aggregates ; larger aggregates were present in the supporting stroma, primarily in dense bands of fibrous connective tissue. The neoplasm was classified as an haemangio-endothelioma. Scintillation analyses of thorium deposits in representative samples of fixed liver tissue yielded gamma activities of 266 $\mu\mu c$ and 189 $\mu\mu c$ thorium-232 per g. and alpha emission activities (zinc sulfide crystal emanations) of 100 $\alpha$ and 40 $\alpha$ per $cm^2$ per hour. Alpha tracts were readily demonstrated in emulsion-dipped autoradiographs of liver (Fig. 13) and spleen (Fig. 14) exposed for 4 weeks.

*Comment* (*Case VI*)

When thorotrast is injected intravenously, the particles are taken up by the reticulo-endothelial system, and the organs which show the greatest concentrations of aggregates of crystal are the liver, spleen, bone marrow and lymph nodes. The use of thorotrast clinically for hepatolienography to visualise the reticulo-endothelial system has resulted in the infrequent induction of primary sarcomas, carcinomas, and mixed neoplasms in the liver. Looney (1960) cited ten cases in the literature of hepatocellular and cholangiocellular carcinoma in patients previously given thorotrast for such radiodiagnostic procedures. Since hepatic carcinomas are associated with other pathological conditions of the liver (*e.g.* cirrhosis), and since in most of these patients thorotrast was administered to diagnose and evaluate hepatic disease, it is difficult to assess the role of pre-cancerous conditions which may have existed at the time of the administration of thorotrast and the extent to which the material may have accelerated the induction of malignancy.

Experimental evidence available to establish a relationship between the induction of primary hepatic neoplasms and thorotrast is concerned with cancers of mesodermal origin. Johansen (1954) produced hepatic reticulo-endotheliomal sarcomas in rabbits ; Selbie (1936) and Guimaraes and Lamerton (1956) have reported similar reticulo-endotheliomas and haemangio-endotheliomas in the liver and spleen of rats and mice. Looney, Hursh, Colodzin and Steadman (1960) described two cases of hepatic sarcomas arising in patients 23 years and 17 years following the intravenous administration of thorotrast. The first was a haemangio-endothelioma, histologically similar to the rare spontaneous Kupffer cell sarcomas (or haemangio-endotheliomas) described by Baker, Paget and Davson (1956) in a series of 28 cases of tumours arising from the sinusoidal endothelium of the liver. The second patient had a multinodular primary hepatic neoplasm, the microscopic description of which was unavailable. Nine other patients with primary hepatic sarcomas developing following thorotrast hepatosplenography have been reported (Looney, 1960) ; the earliest of these malignancies appeared within 38 months of the time of injection. While histologically similar, four were classified as hepato-sarcomas, three as haemangio-epitheliomas, and two as endothelial cell sarcomas.

On the basis of clinical and experimental material available, Looney (1960) suggested that the haemangio-endothelioma was almost a thorotrast-specific tumour. He referred, however, to the occurrence of three similar hepatic tumours in a group of 25 vineyard workers with chronic arsenic poisoning described by Roth (1957) and discussed the controversy whether thorotrast tumours arose from the effects of radiation only, or whether the carcinogenic effect may be related to some extent to the physical presence of the particulate material in the tissue or even to chemical properties of thorium. If these effects are related to tissue damage and repair resulting from radiation from thorium and its daughters, there was no evidence in the studies of Guimaraes and Lamerton (1956) or Bensted and Crookall (1963), who found no indication that tissue destruction of any significance preceded the development of liver neoplasia in rats and mice although the radiation dosage was very large. Selbie (1936) observed fibroblast proliferation at injection sites in the subcutaneous tissues of many of his thorotrast-tumour rats and suggested that neoplasia developed in those animals which gave a vigorous inflammatory reaction to the presence of thorotrast. Clinical reports on the late sequelae of extravasation of thorotrast following intravenous or intra-arterial injection have supported these experimental observations of extensive inflammatory response with the development of marked fibrosis and foreign body granulomas. Bensted and Crookall (1963) found no difference in the incidence of hepatic tumours in a comparison in mice of the late effects of thorotrast and zirconotrast, a non-radioactive colloidal contrast medium. Upton, Furth and Burnett (1956) have studied the effects of radioactive colloidal gold and concluded it was not necessarily the colloidal state of the material which rendered it carcinogenic for the liver. While a number of studies have emphasized the possible role of cirrhosis and related biochemical factors in the livers of thorotrast patients, no recent experimental evidence is available to indicate induction of liver damage and cirrhosis in rats and mice following administration of thorotrast. The picture is further complicated by the report of Ross (1932) of a patient in whom a haemangio-endothelioma in the liver was observed 3 years after a radium needle inadvertently lodged in the septum of the heart. If this rare neoplasm was caused by radiation at a distance from the liver, this would suggest that thorotrast tumours may arise from radiation damage rather than from direct physical or chemical factors.

The haematological studies in Case VI were similar to those of patients in the large series of Looney (1960), and while no predominant picture prevailed in these patients, it was not unusual to find anaemia and an increase in the early forms of the myeloid series. Since bone marrow is a favoured site for thorotrast deposition. destruction of erythropoietic and myelopoietic tissues and the subsequent appearance of circulating immature blood cells could be expected.

### DISCUSSION

### *Radiation dosage in radiotherapy*

The least well documented data on the carcinogenic effects of radiation are those concerned with radiation cancer in man. Relatively few cases of neoplasia which can be directly related to the previous therapeutic irradiation have been described, and these provide only minimum information concerning radiation dose and dose rate, volume of tissue irradiated, and predisposing factors which may have existed at the time of irradiation. Since it is not possible to estimate the total population at risk following radiotherapy, it becomes extremely difficult to relate the small number of cases to frequencies of tumour incidence and radiation exposure.

Inadequate historical data in Cases I to IV prevent precise correlation of sites and incidence of tumours with tissue dose, but these cases support observations that the risk of radiation malignancy is present with doses in the accepted radiotherapeutic range (Table I). The fibrosarcoma of the oesophagus (Case I)

TABLE I.—*Summary of Case Studies of Radiation Induced Cancer*

| Original lesion | Radiation lesion | Age at time of irradiation | Latent period | Estimated Dose |
|---|---|---|---|---|
| I. Carcinoma larynx | Fibrosarcoma, cervical oesophagus | 49 years | 8 years | 4600 r. |
| II. Retinoblastoma | Fibrosarcoma, orbit | 7 months | 4–5 years | 3800 r. |
| III. Hypertrophied lymphoid tissue nasopharynx | Anaplastic carcinoma, lacrimal, maxillary sinus | 12 months | 13 years | see text |
| IV. Tuberculous adenitis | Carcinoma, nasopharynx | 33 years | 31 years | |
| | Carcinoma, larynx | 65 years | 35 years | see text |
| V. Chronic sinusitis | Anaplastic carcinoma, ethmoid sinus | 23 years | 8 years | see text |
| VI. Abdominal pain ?Liver abscess | Haemangio-endothelioma | 19 years | 19 years | see text |

developed in soft tissue after 4900 r., which is within the range of 3000 r.–5000 r reported in a number of clinical studies of osteosarcomas, fibrosarcomas, and chondrosarcomas appearing following radiation therapy for benign or malignant conditions (Cahan *et al.*, 1948 ; Jones, 1953 ; Sabanas, Dahlin, Childs and Ivins,

1956). In the young child with fibrosarcoma in the osseous structures of the orbit (Case II), the determination of radiation dose absorbed in bone is concerned with a number of additional factors. Spiers (1951) has shown that depending upon the radiation quality and the region of absorption within the bone, the radiation dose may be considerably greater than in the soft tissue for the same dose in roentgens. Thus, in the orthovoltage range, for a dose of 2800 r. estimated in the centre of the orbit, the radiation dose absorbed on the surface of the adjacent bone may be of the order of 8500 r. This does appear to represent an excessive dose which could induce radiation malignancy, and raises the important consideration as to whether the tissues of the young child are more susceptible than those of the adult. Simpson and Hempelmann (1957) have reported five cases of osteochondroma in the shoulder in children irradiated in the neck region in infancy ; the minimum skin dose recorded was 125 r.

From radiotherapeutic experience, good clinical response was claimed from irradiation of enlarged tonsils and adenoids, either by X-rays applied externally in small dosage, or by radium applicators introduced into the oral cavity or the nasopharyngeal space. The details of radiation dosage in Case III remain obscure, but since the dosage scheme for external radiotherapy of the order of 50 r. to 100 r. air dose for each exposure was an accepted method, it may be assumed that the tissue dose absorbed would be in the range of 165 r. to 330 r. for " three doses ". While this represents a small radiation dose the data of Simpson *et al.* (1955) and Clark (1955) on children who developed cancer of the thyroid, leukaemia and osteochondromas following previous irradiation to the thymus and neck region with skin doses as low as 125 r. may suggest a greater radiation susceptibility to malignant induction in tissues of infants and young children than in adults. In the past, tuberculous cervical adenitis frequently responded to treatment with small doses of X-rays applied at weekly intervals for some 6 to 8 weeks ; the radiation dosage was of the order of 50 r. to 100 r. in air for each exposure. While no data is available for Case IV, it is probable that she received a tissue dose of at least 400 r. to 750 r. ; radiation scars were observed on her neck from previous therapy. Goolden (1957) and Cade (1957) do not present sufficient data on radiation dose absorbed in those patients who subsequently developed carcinoma of the pharynx and larynx, but draw attention to the frequency of damage to the skin and subcutaneous tissues as an index of radiation dose. While extensive tissue damage has been considered important for the induction of radiation cancer, the range of low doses delivered for benign conditions suggests that no evidence exists for a threshold dose below which radiation carcinogenesis does not occur. The second course of irradiation, however, may have contributed an additional 2000 r. to 4000 r. to the deep structures of the neck, thereby bringing the total radiation dose into the range of cases reviewed by Jones (1953). The evidence suggests that the latent period for the second radiation cancer may be shortened by the additional therapy due to acceleration of existing pre-malignant changes in progress, particularly in view of a history of predisposing conditions of infection and irradiation.

*Radiation dose from thorotrast*

Thorotrast (thorium dioxide sol) is a stable, highly dispersed sol which consists of 24–26 per cent thorium dioxide by volume, 20 per cent dextran, and 0·15 per cent methyl-*p*-hydroxybenzoate as a preservative. The size of the thorium particles range from 3 to 10 $\mu$. The decay series of thorium-232 contains eleven radioactive daughter products (with branching at polonium-216 and bismuth-212); the series ends in stable lead-298. The radionuclides of the series emit alpha, beta or gamma radiations of varying energies; the longest half-life of these is the alpha emission of thorium-232 with $1{\cdot}39 \times 10^{10}$ years.

The inhomogenous radiation produced by alpha emitters, and the non-uniform and patchy anatomical distribution of thorotrast, complicate any attempt at calculation of radiation dosage to the tissue. Correlation with histopathological findings based on terminal burdens is suspect since the uneven and irregular distribution with increasing aggregation and flocculation of thorotrast granules, and migration and redistribution of thorium are constantly changing levels of radiation dose. Furthermore, some of the decay products of the complicated thorium series are soluble and bone-seekers. Thus, average dose to tissue is a meaningless concept, and calculations based on terminal burdens do not necessarily represent the radiation doses which may be responsible for initiating malignant processes.

For hepatosplenography, 75 c.c. of thorotrast injected intravenously was usually given in 25 c.c. amounts daily or every other day. This amount would be the equivalent of 15 g. of thorium dioxide and would represent 2·1 $\mu$g. radium equivalent alpha emission. The energy of emission would be $2{\cdot}5 \times 10^5$ MeV./sec.; radiothorium and other daughter products would eventually build up in a complicated manner into equilibrium at which time the energy emission would be $2{\cdot}2 \times 10^6$ MeV./sec. (Rundo, 1956). The parent thorium-232 is retained almost indefinitely in the reticulo-endothelial system of the liver and spleen; the biological half-time for thorium is of the order of 400 years. Assuming indefinite retention following intravenous administration, some 70–75 per cent would be deposited in the liver, 10–15 per cent in the spleen, and 5–10 per cent in the bone marrow; the estimated average radiation doses would be 1·5, 2·5, and 0·3 rads per week in these organs respectively (Looney, 1960).

Thus, determination of radiation dose rate associated with the development of haemangio-endothelioma is complicated by (1) the non-uniformity of distribution of the radionuclides in the tissue, (2) the intricate decay scheme of thorium-232 and the activities of its daughter products over an extended period, and (3) the effects of geometrical size and shape of the thorium aggregates in the tissue on energy emission and absorption. Following injection, thorotrast flocculates and is deposited in aggregates or granules up to 100 $\mu$ in diameter; this is greater than the ranges in tissue of the alpha particles (the most energetic, polonium-212, has a range of 85 $\mu$) and results in substantial self-absorption. In Case VI, representative samples of liver examined by alpha scintillation counting revealed an emission of $100\alpha$ per cm.$^2$ per hour and $40\alpha$ per cm.$^2$ per hour. Determination of thorium-232 activity in these specimens by gamma spectrometry indicated approximately 270 $\mu$c $^{232}$Th per g. and 190 $\mu$c $^{232}$Th per g. Based on data of Marinelli (1958), and estimates of Rundo (1961), Hursh, Steadman, Looney and Colodzin (1957) and Rotblat and Ward (1956), an approximation of mean radiation dose to the liver following intravenous injection of 75 c.c. of thorotrast ($^{232}$Th and daughters; *RBE* = 4–10) would be 100 rads per year and an estimated mean accumulated dose in the order of 8000 to 10,000 rads after 19 years. Rundo (1961) suggested an extra 5 to 10 per cent contribution from beta particles and 2 to 5 per cent from gamma rays. This agrees with average estimated radiation dosages to the liver of the order of 700 to 15,000 rads in patients with terminal body burdens of thorium-232 studied by these authors.

*Predisposing Factors*

The data available from clinical experience provide little material to understand the role of predisposing factors in the mechanisms of radiation carcinogenesis. A pattern emerges, however, which suggests that (1) the doses of radiation required to induce certain tumours were of the order of thousands of roentgens and (2) the doses were of a magnitude that produced severe tissue damage since, in almost all cases studied, it appeared that extensive tissue damage preceded the appearance of cancer. Certain inflammatory processes, e.g., tuberculosis, osteitis, etc., appear to enhance the carcinogenic effect of irradiation. Since frequently protracted periods of treatment to relatively high doses are necessary in radiotherapy of malignant disease, it may be the radiation itself which supplies the inflammatory factor by the stromal response in those instances where evidence of previous inflammation is lacking. These observations suggest that the type and degree of tissue damage induced may be more important than the radiation dose, and that neoplasia may develop in regenerating tissue during processes of repair.

*Latent periods*

The controversial concept of latent interval applied to experimental studies has been criticised for lack of precise definition of mechanisms which are operating between the time of administration of radiation and the earliest identification of induced neoplasia. Clinically, however, a latent interval may be defined as the period of time between exposure to radiation (or the last treatment cycle in radiotherapy) and the development of clinical cancer. For Cases I to VI, the latent intervals varied between 4 and 34 years. This period has little meaning, however, in the thorotrast patients, where in the course of the disintegration of thorium and its daughter elements, alpha, beta, and gamma radiations are continuously emitted from the thorium deposits in the reticulo-endothelial tissues. In addition, difficulty arises to determine suitable latent intervals in those patients whose treatment extended over several months or years, and in those instances where two or more courses of therapeutic radiation were separated by many years. While clinical latent periods are difficult to ascertain, they definitely appear to be reduced with higher doses of radiation and where tissues have been irradiated in the presence of inflammatory disease.

Insufficient human data are available to correlate latent intervals with age at the time of irradiation, and sites and types of tumour induction. Jones (1953 estimated a mean latent interval of about 9 years for irradiation sarcomas in bone where the doses reported were some 3000 r. to 5000 r. or more. Cases I and II, both irradiation sarcomas, fit into this group with relatively short latent intervals. In the series of Goolden (1957), the latent interval range of 10 to 35 years (mean 25·5 years) for radiation carcinomas in the pharynx may suggest that the interval for the development of carcinoma could be greater where small radiation doses are involved. Cases III and IV would support these findings. The clinical intervals for the thorotrast patients fall between these two extremes, and while sarcomas and carcinomas result from the internal fixation of this radionuclide, it is interesting that the interval range and mean period for induction of both types of neoplasia in the two distinct clinical groups of patients are almost identical (Table II).

TABLE II.—*Latent Interval for Thorotrast Patients*

| Neoplasm | Number of Patients | Reference | Range (years) | Mean (years) |
|---|---|---|---|---|
| Carcinoma of maxillary sinus | 10 | Feldman *et al.* (1963) Case V | 10–20 | 13·6 |
| Haemangio-endothelioma | 9 | Looney (1960) Case VI | 8–22 | 15·4 |

CONCLUSIONS

The problem of the incidence of cancer following the medical use of radioactive materials is receiving considerable attention. Bone-seeking radionuclides, particularly alpha-emitters, formed in nuclear energy processes are being studied to determine possible carcinogenic properties and to learn about the processes of tumour induction with these materials. Thorotrast tumour patients represent important clinical material to evaluate and compare the late effects of continuous low level irradiation and to provide information on the biological mechanisms of radiation carcinogenesis in man. Although 62 cases of thorotrast cancer have been reported, there are, as yet, no comprehensive statistics on incidence, dosage, or usage. Thus, any additional data on previous material or on new cases represent important and substantial contributions to our scanty knowledge of the biological actions of internally deposited radionuclides, and particularly for the study of the effects of chronic irradiation by alpha particles in man. Thorotrast has been used extensively in diagnostic radiology, and it is expected that an increase in clinical material will become available which may be studied by epidemiological methods as advocated by Marinelli (1961). In addition, while these patients do not contribute significantly to information on genetic dose in the population, they do represent available cases for clinical studies to further our understanding of maximum permissible levels of body burden for radionuclides.

The hazards to patients from diagnostic procedures in radiology are extremely small. Stewart, Webb, and Hewitt (1958) claim that prenatal obstetrical radiodiagnostic examination may be related to the subsequent development of leukaemia or other cancers in the child. Further extensive studies to establish relationships between such diagnostic exposures and childhood malignancies are required. Although human data from radiotherapy, radium ingestion and thorotrast administration suggest that tumour production is a rare sequel of radiation exposure, particularly for dose ranges such as those used in radiotherapy, it is possible that therapeutic radiation by megavoltage apparatus and artificial radioisotopes may result in a further increase of radiation cancer incidence in the future. A certain morbidity must be accepted if we are to derive the benefits of accurate radiological diagnosis and effective treatment of malignant disease with radiation. The very small risk of radiation cancer should never become a deterrent to the medical uses of radiation, since the elimination of all risk would apply equally to all therapeutic measures, radiation or otherwise. The remote danger of producing a new tumour by radiation has to be accepted where this form of treatment constitutes the only hope and practical and reliable means of eliminating an existing malignancy and thus preserving or prolonging the life of the patient.

The authors wish to thank Professors W. V. Mayneord and L. F. Lamerton of the Department of Physics, Institute of Cancer Research, Dr. Russell H. Morgan, Chairman of the Department of Radiology, The Johns Hopkins Hospital, and Dr. Wiley D. Forbus, former Chairman of the Department of Pathology, Duke University School of Medicine, for their interest and encouragement.

J. I. F. is an Advanced Fellow in Academic Radiology of the James Picker Foundation.

### REFERENCES

Ashton, N —(1958) 'Cancer.' Edited by R. Raven ; London (Butterworths) vol. 2, p. 599.
Aub, J. C., Evans, R. D., Hempelmann, L. H. and Martland, H. S.—(1952) *Medicine*, **31**, 221.
Baker, H. de C., Paget, G. E. and Davson, J.—(1956) *J. Path. Bact.*, **72**, 173.
Bensted, J. P. M., Blackett, N. M. and Lamerton, L. F.—(1961) *Brit. J. Radiol.*, **34**, 160.
*Idem* and Crookall, J. O.—(1963) *Brit. J. Cancer*, **17**, 62.
Cade, S.—(1957) *Brit. J. Radiol.*, **30**, 393.
Cahan, W. G., Woodward, H. Q., Higginbotham, N. L., Stewart, F. W. and Coley, B. L.—(1948) *Cancer*, **1**, 3.
Clark, D. E.—(1955) *J. Amer. med. Ass.*, **159**, 1007.
Cruz, M., Coley, B. L. and Stewart, F. W.—(1957) *Cancer*, **10**, 72.
Duffy, B. J. and Fitzgerald, P. J.—(1950) *Ibid.*, **3**, 1018.
Feldman, F., Seaman, W. B. and Wells, J. S.—(1963) *Amer. J. Roentgenol.*, **89**, 1147.
Glucksmann, A., Lamerton, L. F. and Mayneord, W. V.—(1957) 'Cancer.' Edited by R. Raven ; London (Butterworths) vol. 1, p. 497.
Goolden, A. W. G.—(1951) *Brit. med. J.*, ii, 1110.—(1957) *Brit. J. Radiol.*, **30**, 626.
Gros, C. M., Fruhling, L. and Keiling, R.—(1955) *Bull. Ass. franç Cancer*, **42**, 556.
Guimaraes, J. P. and Lamerton, L. F.—(1956) *Brit. J. Cancer*, **10**, 527.
Hofer, O.—(1952) *Dtsch. Zahnärztl. Z.*, **7**, 736.
Holinger, P. H. and Rabbatt, W. F.—(1953) *Laryngoscope*, **63**, 105.
Hursh, J. B., Stedman, L. T., Looney, W. B. and Colodzin, M.—(1957) *Acta radiol.*, **47**, 481.
Johansen, C.—(1954) *Acta path. microbiol. scand.*, Suppl. 92.
Jones, A.—(1953) *Brit. J. Radiol.*, **26**, 273.
Kligerman, M., Lattes, R. and Rankow, R.—(1960) *Cancer*, **13**, 967.
Looney, W. B.—(1960) *Amer. J. Roentgenol.*, **83**, 163.
*Idem* and Colodzin, M.—(1956) *J. Amer. med. Ass.*, **160**, 1.
*Idem*, Hursh, J. B., Colodzin, M. and Stedman, L. T.—(1960) *Acta Un. int. Cancr.*, **16**, 435.
Marinelli, L. D.—(1958) *Radiology*, **70**, 93.—(1961) *Proc. int. Congr. Radiol.*, **9**, 1234.
Pettit, V. D., Chamness, J. T. and Ackerman, L. D.—(1954) *Cancer*, **7**, 149.
Raventos, A., Gross, S. W. and Pendergrass, E. P.—(1960) *Amer. J. Roentgenol.*, **83**, 145.
Ross, J. M.—(1932) *J. Path. Bact.*, **35**, 899.
Rotblat, J. and Ward, G.—(1956) *Phys. Med. Biol.*, **1**, 125.
Roth, F.—(1957) *Z. Krebsforsch.*, **61**, 468.
Rundo, J.—(1956) *Phys. Med. Biol.*, **1**, 138.—(1961) *Proc. int. Congr. Radiol.*, **9**, 1258.

SABANAS, A. O., DAHLIN, D. C., CHILDS, D. S. AND IVINS, J. C.—(1956) *Cancer*, **9**, 528.
SELBIE, F. R.—(1936) *Lancet*, ii, 847.
SIMPSON, C. L. AND HEMPELMANN, L. H.—(1957) *Cancer*, **10**, 42.
*Iidem* AND FULLER, L. M.—(1955) *Radiology*, **64**, 840.
SKOLNICK, E. M., FORNATTO, E. J. AND HEYDEMANN, J.—(1956) *Ann. Otol., etc., St. Louis*, **65**, 915.
SOM, M. L. AND PEIMER, R.—(1955) *Arch. Otolaryng., Chicago*, **62**, 428.
SPIERS, F. W.—(1951) *Brit. J. Radiol.*, **24**, 365.
STEWART, A., WEBB, J. AND HEWITT, D.—(1958) *Brit. med. J.*, i, 1495.
WILSON, E. H. AND ASPER, S. P. Jr.—(1960) *A.M.A. Arch. intern. Med.*, **105**, 244.
UPTON, A. C., FURTH, J. AND BURNETT, W. T. Jr.—(1956) *Cancer Res.*, **16**, 211.